# HLA class II-Restricted CD8+ T cells in HIV-1 Virus Controllers

**DOI:** 10.1038/s41598-019-46462-8

**Published:** 2019-07-15

**Authors:** Tinashe E. Nyanhete, Alyse L. Frisbee, Todd Bradley, William J. Faison, Elizabeth Robins, Tamika Payne, Stephanie A. Freel, Sheetal Sawant, Kent J. Weinhold, Kevin Wiehe, Barton F. Haynes, Guido Ferrari, Qi-Jing Li, M. Anthony Moody, Georgia D. Tomaras

**Affiliations:** 1 Duke Human Vaccine Institute, Duke University School of Medicine, Durham, NC 27710 USA; 2 Department of Immunology, Duke University School of Medicine, Durham, NC 27710 USA; 3 Department of Medicine, Duke University School of Medicine, Durham, NC 27710 USA; 4 Department of Molecular Genetics and Microbiology, Duke University School of Medicine, Durham, NC 27710 USA; 5 Department of Surgery, Duke University School of Medicine, Durham, NC 27710 USA; 6 Department of Pediatrics, Duke University School of Medicine, Durham, NC 27710 USA; 7 Present Address: University of Virginia Department of Microbiology, Immunology and Cancer Biology, 345 Crispell Drive, University of Virginia Health System, Charlottesville, Virginia 22908 USA

**Keywords:** HIV infections, Antigen presentation, Infection, Lymphocyte activation

## Abstract

A paradigm shifting study demonstrated that induction of MHC class E and II-restricted CD8+ T cells was associated with the clearance of SIV infection in rhesus macaques. Another recent study highlighted the presence of HIV-1-specific class II-restricted CD8+ T cells in HIV-1 patients who naturally control infection (virus controllers; VCs). However, questions regarding class II-restricted CD8+ T cells ontogeny, distribution across different HIV-1 disease states and their role in viral control remain unclear. In this study, we investigated the distribution and anti-viral properties of HLA-DRB1*0701 and DQB1*0501 class II-restricted CD8+ T cells in different HIV-1 patient cohorts; and whether class II-restricted CD8+ T cells represent a unique T cell subset. We show that memory class II-restricted CD8+ T cell responses were more often detectable in VCs than in chronically infected patients, but not in healthy seronegative donors. We also demonstrate that VC CD8+ T cells inhibit virus replication in both a class I- and class II-dependent manner, and that in two VC patients the class II-restricted CD8+ T cells with an anti-viral gene signature expressed both CD4+ and CD8+ T cell lineage-specific genes. These data demonstrated that anti-viral memory class II-restricted CD8+ T cells with hybrid CD4+ and CD8+ features are present during natural HIV-1 infection.

## Introduction

There are nearly 37 million people living with HIV-1, and an estimated 1.8 million new HIV-1 infections were reported in 2017^1^. Although combination antiretroviral therapy (ART) has helped to effectively control HIV-1 replication, prevent the development of AIDS, prolong life and reduce the risk of transmission, there are a still a number of limitations involved in providing this lifelong therapy to people living with HIV-1^2^. These limitations, which include drug resistance, drug toxicity, limited lifelong patient adherence, maintenance of a latent HIV-1 reservoir and the economic costs of providing drugs to resource poor nations (which account for over 70% of people living with HIV-1), highlight the need to identify an effective means of controlling the virus in the absence of ART^1,2^. As a result, large-scale efforts and resources are being directed towards vaccine research and development.

The hallmark of an effective viral vaccine is to elicit robust, effective and extensive humoral and cellular immune responses against the virus. Within the context of HIV-1, broadly neutralizing antibodies (bNabs) that prevent infectivity, non-neutralizing antibodies with Fc-mediated antiviral activity, and cytotoxic CD8+ T cells that eliminate virus-infected cells have emerged as three major elements that regulate viral clearance^3–8^. Optimism for T cell-based vaccines received a major boost with the report of a non-human primate (NHP) T cell-based vaccine study that resulted in the induction of a novel subset of MHC class II and class E-restricted CD8+ T cells^9,10^. These novel subsets of CD8+ T cells were associated with complete clearance of SIV in 50% of animals^11,12^. Surprisingly, the majority of the epitopes targeted by these protective CD8+ T cells were MHC Class II-restricted and the remainder were MHC-E restricted; challenging the conventional MHC-I restricted CD8+ T cell paradigm^9,13^. These findings were remarkable because natural infection with HIV-1 or SIV usually induces T cell responses that decrease virus replication in acute infection but fail to eradicate the virus^13,14^. This work highlights a potential role for unconventional MHC class II-restricted CD8+ T cells in controlling viremia. Further analysis of elicitation of these atypical CD8+ T cells in humans by other pathogens will elucidate whether they are an attractive target for induction by vaccine strategies.

Although rarely reported, the presence of unconventional MHC class II-restricted CD8+ T cells has been reported in mice and human studies^15–23^. CD4-deficient, MHC class II-deficient mice or CD4-deficient mice with a transgenic class II-specific T Cell Receptor (TCR) provide the most evidence for unconventional MHC class II-restricted CD8+ T cells elicited by either bacterial or viral infections in mice^15–19^, although there is some evidence for very low frequency unconventional CD8+ T cells in wild-type B6 mice^18^. In humans, HLA class II-restricted CD8+ T cells were previously observed in human transplant recipients who generated allo-reactive CD8+ T cells^20–23^, indicating a possible role of these unconventional cells in allo-recognition. It has been suggested that the highly inflammatory and immunogenic conditions following transplantations might be involved in the misdirected lineage instruction of these HLA class II-restricted CD8+ T cells^20^. In the context of viral infection, cytolytic effector memory HIV-1-specific class II-restricted CD8+ T cells have also recently been observed in a small subset of HIV-1 virus controller (VC) patients who naturally control viremia in the absence of any form of therapy^24^. However, it is still not clear whether this unique cell population is present in any other cohorts. Given these infrequent observations of HLA class II-restricted CD8+ T cells in humans, it is important to identify other cohorts with these cellular subsets, to elucidate their functional role and investigate the factors responsible for the induction of these cells. In addition, due to their association with HIV-1 clearance in non-human primates, it is especially important to further investigate the functional role of HLA class II-restricted CD8+ T cells in the setting of HIV-1 infection.

HIV-1 VCs represent a rare subset of HIV-1-infected individuals (<1%) that can maintain undetectable HIV-1 RNA levels without therapy^25^. We and others have previously shown that CD8+ T cells from VCs have an enhanced ability to inhibit HIV-1 replication in infected autologous CD4+ T cells through both soluble anti-viral factors and contact dependent, target cell killing^14,26–31^. We have also shown that this potent VC CD8+ T cell antiviral activity is augmented by enhanced mRNA stability and transcription of antiviral cytokines^30,31^, and also the presence of multifunctional and highly proliferative HIV-1 specific CD4+CD8+ double positive T cells^32^. Thus, the VC patient cohort provides us with a human model of HIV-1 virus replication suppression that we can use to study CD8+ T cell mediated suppression of HIV-1 and also investigate the presence and functional role of HLA class II-restricted CD8+ T cells in HIV-1+ patients.

The aim of this study was to determine the presence of unconventional class II-restricted CD8+ T cells in a southern U.S. HIV-1 virus controller cohort and to define their anti-viral gene expression signature and relationship to the CD4+ T cell lineage. We first determined the frequency, memory phenotype and viral suppressive ability of these HLA class II-restricted CD8+ T cells in different cohorts (seronegative healthy donors, VCs and chronic patients) to understand whether different disease states affected induction/maintenance, frequency and memory phenotype of these cells. Using *ex vivo* cells from HIV-1 VCs with a consistent presence of class II-restricted CD8+ T cells at multiple time points during the course of infection, we examined the nature of these rare cells through analysis of their anti-viral gene expression signature, TCR repertoire diversity, and expression of T cell lineage-specific transcription factors representative of ontogeny. These findings define the presence of unconventional anti-viral HIV-1 Gag-specific class II-restricted CD8+ T cells with a distinct transcriptional profile characterized by the expression of both CD4 and CD8-lineage specific genes.

## Results

### Primary human CD8+ T cells can inhibit virus replication through both HLA Class I and Class II recognition

**Figure 1:**
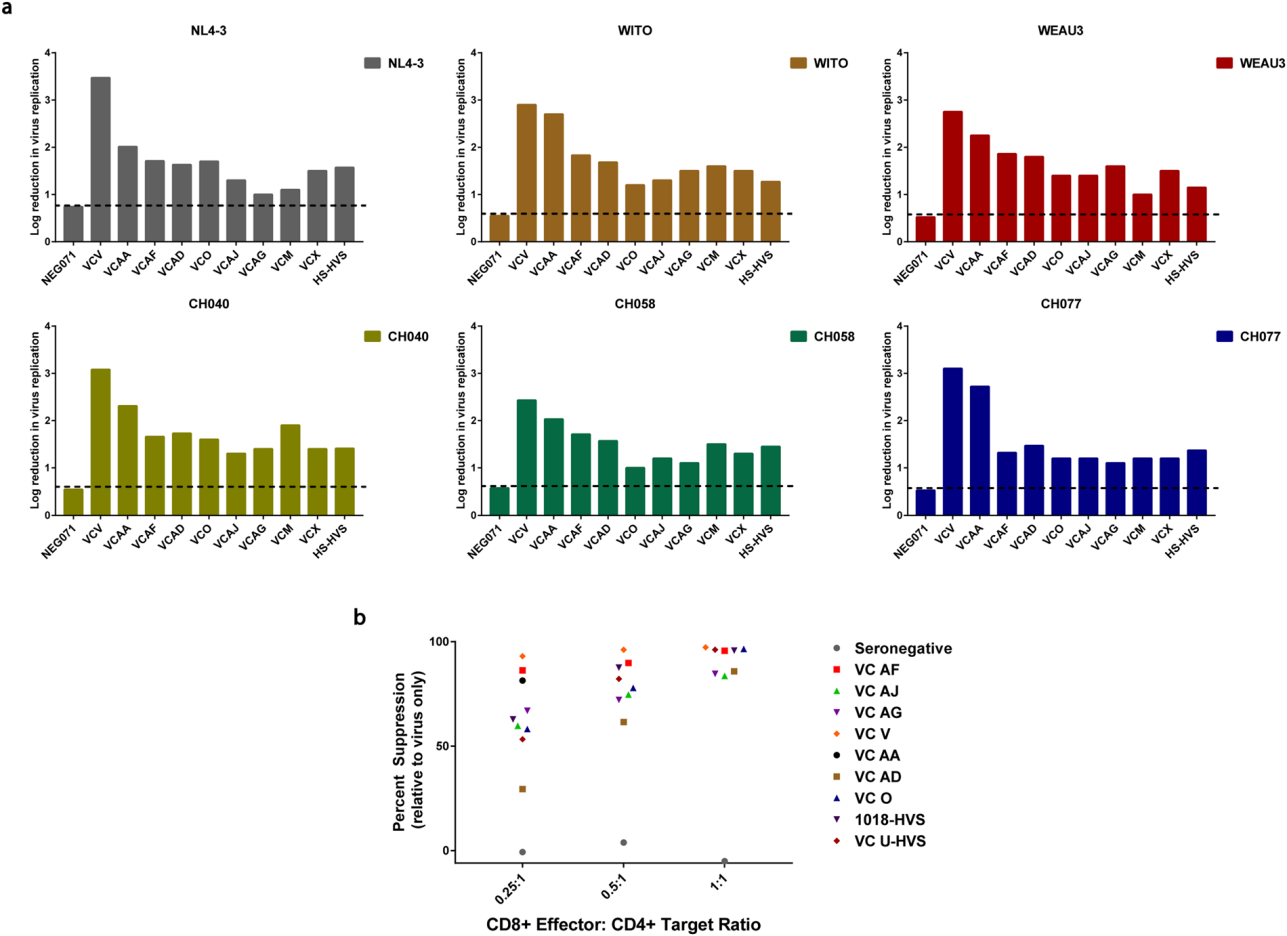
(**A**). HIV-1 VC CD8+ T cells suppress replication of multiple clade B viruses. CD8+ T cells isolated from nine VC patients, one VC CD8+ T cell line (HS-HVS) and one seronegative donor (NEG071) were tested for antiviral activity against a panel of clade B lab-adapted and T/F viruses in the CD8 VIA. Autologous CD8 and CD4 T cells were co-cultured together in duplicate at 4 different Effector:Target ratios (0.25:1, 0.5:1, 1:1 and 2:1), with the highest Effector:Target ratio (2:1) shown. Virus suppression is expressed as a log reduction in virus replication in the presence of CD8+ T cells compared to target cell only control at the 2:1 Effector:Target ratio. The dotted black horizontal line is the cut-off for positive log reduction in virus replication and is determined using the NEG071 negative control. VC O and VC AF were run on 2 separate days to confirm reproducibility of the assay. (**B**) Dose dependent CD8+ T cell inhibition of clade B matched T/F infectious molecular clone CH058.c. CD8+ T cells isolated from seven VC patients, two VC CD8+ T cell lines (VCU-HVS and 1018-HVS) and one seronegative donor were tested for antiviral activity against the T/F virus CH058.c in the CD8 VIA. Autologous CD8 and CD4 T cells were co-cultured together at 0.25:1; 0.5:1; and 1:1 Effector:Target ratios. Percent suppression on the y-axis represents virus replication suppression as a percentage using changes in relative light units (RLU) from wells containing CD8+ T cells compared with control wells with virus only (lacking CD8+ T cells). Each VC was considered as a biological replicate due to limited availability of these rare patient samples. VCAA displayed high levels of viral replication suppression in infected target autologous CD4+ T cells at the lowest ratio tested (0.25:1) so no further testing was done at higher ratios (0.5:1 and 1:1) due to limited VCAA sample availability.

In an effort to investigate the possible functional role of HLA class II-restricted CD8+ T cells in HIV-1 viral control, we chose to look at the nature of the potent anti-HIV-1 CD8+ T cell responses in HIV-1 VCs with broad CD8+ T cell mediated anti-HIV-1 inhibitory activity^14,26,30,31^. VC patients with a viral load below 5,000 copies/mL and a CD4+ T cell count above 400 cells/µL (Table 1) were enrolled for this study. The potency and breadth of CD8+ T cell-mediated virus inhibition was first assessed using a contact-mediated viral inhibition assay (VIA) against a panel of lab-adapted (NL4-3) and full-length subtype B (WITO, WEAU3, CH040.c, CH058.c and CH077) transmitted/founder virus strains^33^. Primary CD8+ T cells isolated from the peripheral blood of the VC patients were tested for HIV-1 inhibition in HLA-matched primary autologous CD4+ enriched T cells. While the magnitude of inhibitory activity varied, all the VCs tested possessed broad CD8+ mediated viral inhibitory activity against the panel of HIV-1 viruses (Fig. 1A). We next tested whether CD8+ T cell mediated HIV-1 antiviral activity was dependent on the concentration of CD8+ T cells. Anti-viral activity increased with increasing effector (CD8+): target (CD4+) ratios with a subtype B T/F virus CH058.c (Fig. 1B). The primary CD8+ T cells isolated from seronegative donors lacked antiviral activity (Fig. 1B). These results confirmed the potent HIV-1 specific antiviral response within the CD8+ T cell population of HIV-1 VC patients.

**Table 1:** HIV-1 Virus Controller Cohort.

Patient	HLA Allele	CD4 Cell Count (CELLS/µL)	Viral Load (COPIES/ML)	ART
VC M	DRB1*0701	719–966	1020–1035	Naive
VC O	DQB1*0501	834	1320	Naive
VC V	DRB1*0701	1269	67	Naive
VC X	DRB1*0701	565–717	601–2660	Naive
VC AA	DRB1*0701	462–581	<20–547	08/2013
VC AD	DRB1*0701	407–524	716–1040	Naive
VC AF	DRB1*0701	729–1102	<20–47	Naive
VC AG	DQB1*0501	1420	47	Naive
VC AH	DRB1*0301	1182–1899	<20–392	Naive
VC AJ	DQB1*0501	493	108	Naive
VC AX	DRB1*0701	1281	<20	Naive

List of HIV-1 VC patients used in the study. VC AA went onto ART after enrollment into the study, and hence time points before and after ART enrollment were used when determining the frequency and memory phenotype of class II-restricted CD8+ T cells.

**Figure 2:**
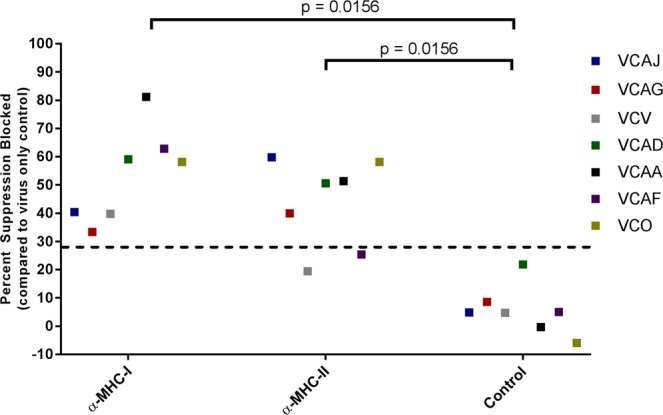
HIV-1 VC HLA Class I and HLA Class II-restricted CD8+ T cell-mediated inhibition of T/F virus replication in autologous CD4+ T cells. Autologous CD8+ T cells isolated from seven VCs were tested for blocking of CD8 antiviral activity by monoclonal antibodies to MHC molecules. 5 µg/ml of the MHC blocking antibodies was used to block the MHC molecules before the infected CD4+ T cells were co-cultured with the CD8+ T cells. The CD8+ T cells were co-cultured with the infected autologous CD4+ T cell targets at a range of Effector:Target ratios of 0.25:1, 0.5:1 and 1:1, with results for the 0.25:1 co-culture ratio shown in the figure. The capacity of each monoclonal antibody to block CD8+ T cell mediated antiviral activity is represented as percent CH058.c virus replication suppression blocked in autologous CD4+ T cells in the presence of either the anti-MHC mAb or an isotype negative control Ab. The cutoff for positive percent suppression blocked is 28.0% (calculated as the Mean+ (3× standard deviation)). Sign test (p-value < 0.05) provided evidence of a statistically significant difference in the paired response between MHC-I or MHC-II and negative control in the blocking assays. Assay reproducibility was confirmed by 2 independent operators who both assayed VC AF and VC AJ, while VC V and VC O were run on 2 separate days by the same operator to further confirm assay reproducibility.

To determine whether HLA class II-restricted CD8+ T cells are part of the anti-HIV-1 cellular repertoire in chronic virus controllers, we interrogated a VC cohort (Table 1) for the presence of HLA class II-restricted CD8+ T cell antiviral activity capable of suppressing T/F virus CH058.c replication in autologous CD4+ T cells. An MHC blocking virus inhibition assay (VIA) was used in which infected primary CD4+ T cells were treated with either 3F10 (anti-MHC class I monoclonal antibody) or L243 (anti-MHC class II monoclonal antibody) (Supp Fig. 1) prior to incubation with autologous primary CD8+ T cells. Infection of CD4+ T cells resulted in a ~15% drop in their viability. Infected CD4+ T cell viability was not affected by the addition of the L243 and 3F10 monoclonal antibodies (Supp Fig. 2A). We found that HLA class II-restricted CD8+ T cells, capable of suppressing T/F virus CH058.c replication, were present in five out of the seven HIV-1 VC patients tested (Fig. 2). In addition to HLA class II-restricted antiviral CD8+ T cell activity, all VC subjects possessed HLA class I-restricted antiviral activity, as expected.

To test whether contact was required for the MHC blocking activity, the targets and effector cells from VC AH were separated in a transwell MHC blocking VIA (Supp Fig. 2B). Class II-blocking was abrogated, consistent with a contact dependent mechanism (Supp Fig. 2B). These data indicated that human HIV-1 VC subjects possessed HIV-1 specific HLA class II-restricted specific CD8+ T cells capable of suppressing HIV-1 replication in autologous CD4+ T cells.

### Detectable low frequency of memory HLA DRB1*0701 restricted Gag_293-312_–specific CD8+ T cells in HIV-1 VC patients

We next sought to determine the frequency and memory phenotype of the HLA class II-restricted CD8+ T cells in HIV-1 VCs (Table 1) and also in chronic patients (Table 2) and seronegative healthy donors (Table 3).

**Table 2:** HIV-1+ Chronic Patient Cohort.

Patient	HLA ALLELE	CD4 Cell Count (cells/µL)	Viral Load (copies/mL)	Geographic Region
425	DRB1*0701	485	17010–367500	Malawi
153	DRB1*0701	823	21665–58573	USA
830	DRB1*0701	424	16108–27075	South Africa
432	DRB1*0701	253	17010	Malawi

List of HIV-1+ chronic patients used in the tetramer-staining assay.

**Table 3:** Seronegative (healthy) donor cohort.

Patient	HLA Allele	Geographic Region
091	DRB1*0701	USA
242	DRB1*0701	USA
653	DRB1*0701	USA
736	DRB1*0701	USA
918	DRB1*0701	USA

List of Healthy donors (seronegatives) used for tetramer staining assays in this study.

We, and others, have previously shown that VCs and chronically infected HIV-1 donors (chronic patients) with anti-viral CD8+ activity contain predominantly Gag-specific effector CD8+ T cells^30,34–40^. Within the HIV-1 Gag-p24 region is the immune-dominant, HLA class II-restricted epitope spanning from amino acid position 293–312, Gag_293-312_, that is targeted in more than 90% of HIV-1 VCs and treated patients^41^. Thus, given the importance of Gag-specific antiviral activity, we utilized the available DRB1*0701 Gag_293-312_ -specific class II tetramer in those patients who were HLA DRB1*0701 positive to assess the prevalence of Gag-specific class II-restricted CD8+ T cells in HIV-1 infected subjects.

In this VC cohort, four of the seven VC patients tested in the MHC blocking experiment (VC V, VC AA, VC AD and VC AF) HLA matched the available HLA class II Gag_293-312_ tetramer (DRB1*0701), and of these four patients only two (VC AA and VC AD) demonstrated blockable HLA class II-restricted CD8+ T cell activity (Fig. 2). Two VCs (VC M and VC X) that had demonstrated CD8+ T cell-mediated antiviral activity against a panel of viruses (Fig. 1A) and one additional VC within the cohort (VC AX) that had not been previously screened for CD8+ T cell mediated antiviral activity were also DRB1*0701 tetramer matched and thus included in the tetramer analysis (Table 1). The seven DRB1*0701 tetramer matched VC patients (VC M, VC V, VC X, VC AA, VC AD, VC AF and VC AX) had PBMC samples from at least 2 time-points, while the available chronic viremics and seronegative donors had PBMC samples from only one time-point due to limited sample availability.

**Figure 3:**
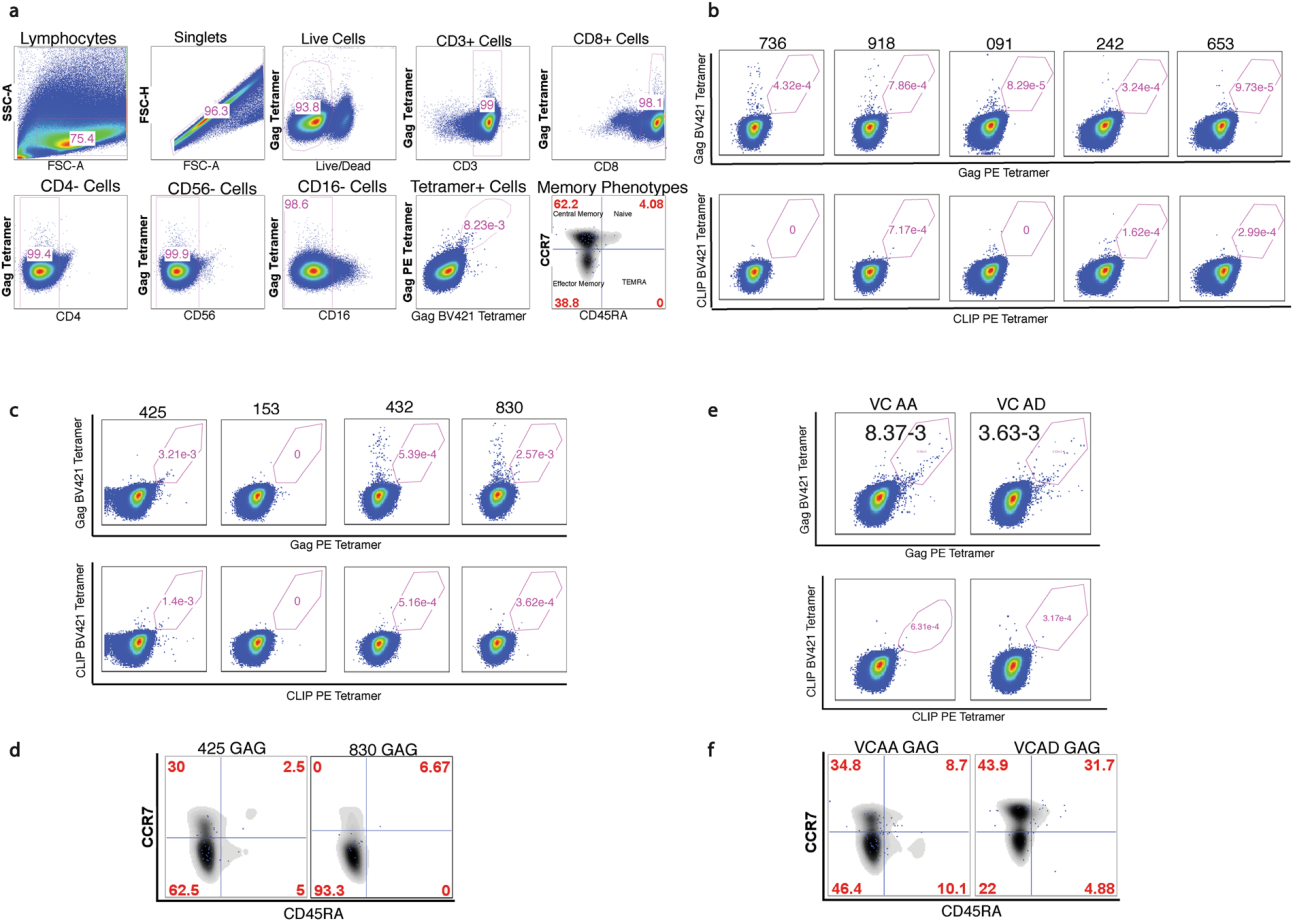
(**A**). Gating strategy for flow cytometry analysis of tetramer-positive HLA DRB1-restricted Gag-specific CD8+ T cells in HIV-1 in our patient cohorts. Cells were first gated for lymphocytes (SSC-A vs. FSC-A) and singlets (FSC-H vs. FSC-A). The single cell lymphocytes were then stained with Live/Dead Aqua stain to isolate the live cells. The live cells were further analyzed for expression of CD3 and CD8 while gating out CD4+ cells, CD56+ and CD16+ cells (Natural Killer cells) so as to narrow down the target population to live, healthy CD8+ T cells (CD3+, CD8+, CD4−, CD56−, CD16−). Gag or CLIP tetramer positive cells were then analyzed from the pure CD8+ T cell fraction by double fluorochrome verification (PE and BV421) to ensure discrimination of true tetramer binding events. Only events that were double positive for the PE and BV421 tetramers were considered to be the tetramer positive events. The memory phenotypes of the tetramer positive events were defined by the surface expression of CCR7 and CD45RA. Naïve tetramer positive events were double positive for CCR7 and CD45RA. Central Memory tetramer positive events were CCR7+ and CD45RA-. Effector Memory tetramer positive events were CCR7- and CD45RA-. Terminally differentiated effector memory (TEMRA) tetramer positive events were CCR7- and CD45RA+ ^44^. (**B**) Gag-specific HLA DRB1*0701 restricted CD8+ T cells are absent in HLA-matched seronegative healthy individuals. Anti-CD3/CD28 activated PBMCs were enriched for CD8+ T cells by negative selection after seven days in culture and subsequently stained with PE and BV421-labeled Gag_293-312_ tetramers and analyzed by flow cytometry. The human CLIP_87-101_ –DRB1 tetramer served as the negative control. Results are presented as a percentage of the CD8+ T cells in the PBMC pool. DRB1-restricted HIV-1 Gag_293-312_ –reactive CD8+ T cells are absent in activated PBMCs from healthy donors. (**C**) Gag-specific HLA DRB1*0701 restricted CD8+ T cells are rarely detectable in HLA-matched HIV-1 Chronic patients. Anti-CD3/CD28 activated PBMCs were enriched for CD8+ T cells by negative selection after seven days in culture and subsequently stained with PE and BV421-labeled Gag_293-312_ tetramers and analyzed by flow cytometry. The human CLIP_87-101_ –DRB1 tetramer served as the negative control. Results are presented as a percentage of the CD8+ T cells in the PBMC pool. DRB1-restricted HIV-1 Gag_293-312_ –reactive CD8+ T cells are barely detectable in activated PBMCs from HIV-1+ Chronic patients. (**D**) The majority of DRB1-restricted Gag_293-312_ specific CD8+ T cells reside in the effector memory (CCR7^−^CD45RA^−^) subsets in PBMC in the HIV-1+ Chronic patients. DRB1-restricted Gag_293-312_ specific CD8+ T cells from chronic patients 425 and 830 (2 chronic patients with barely detectable DRB1-restricted Gag_293-312_ specific CD8+ T cells) were separated into four subsets (Naïve, Central Memory, Effector Memory and Terminally-differentiated Effector Memory) based on CD45RA and CCR7 surface expression levels. (**E**) Gag_293-312_ tetramer detection of Gag_293-312_ -specific HLA-DRB1-restricted CD8+ T cells in VCAA and VCAD. Anti-CD3/CD28 activated PBMCs were enriched for CD8+ T cells by negative selection after seven days in culture and subsequently stained with PE and BV421-labeled Gag_293-312_ tetramers and analyzed by flow cytometry. The human CLIP_87-101_ –DRB1 tetramer served as the negative control. Results are presented as a percentage of the CD8+ T cells in the PBMC pool. DRB1-restricted HIV-1 Gag_293-312_ –reactive CD8+ T cells can be readily detected in activated PBMCs from anHIV-1virus controller individuals. (**F**) The majority of the DRB1-restricted Gag_293-312_ specific CD8+ T cells in VC AA and AD reside in the effector memory (CCR7^−^CD45RA^−^) and central memory (CCR7^+^CD45RA^−^) subsets in PBM**Cs**. DRB1-restricted Gag_293-312_ specific CD8+ T cells from VC AA and VC AD were separated into four subsets (Naïve, Central Memory, Effector Memory and Terminally-differentiated Effectors) based on CD45RA and CCR7 surface expression levels.

Since memory HIV-1-specific CD8+ T cells are known to be important for controlling viral replication^42,43^, we examined the frequency and memory phenotype of HLA DRB1-restricted CD8+T cells. CCR7+ CD45RA+ cells define naïve T cells, CCR7+CD45RA− define a central memory phenotype, CCR7−CD45RA+ define a terminally differentiated effector memory phenotype, and CCR7−CD45RA− cells define an effector memory phenotype^44^. To increase specificity of the class II staining, we utilized a dual staining approach (Fig. 3A) with different fluorophores for HIV-1 Gag and negative control tetramer presenting the invariant peptide, CLIP^45^.

**Figure 4:**
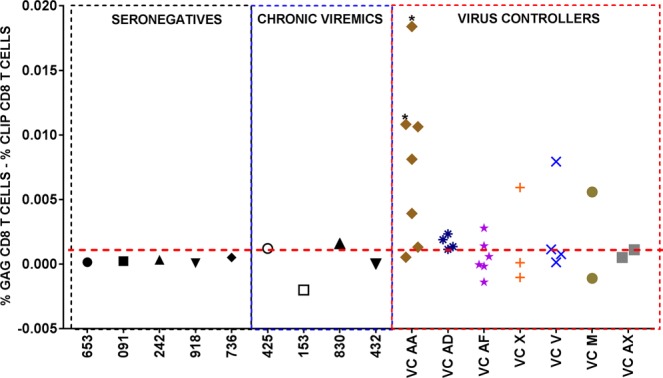
Gag-specific HLA DRB1*0701 restricted CD8+ T cells are present in multiple VC patients at different draw dates. PBMCs obtained from VC patients at different draw dates were screened for the presence of Gag-specific HLA class II-restricted CD8+ T cells using tetramer staining. The frequency of CLIP+ CD8+ T cells was subtracted from the frequency of Gag+ CD8+ T cells so as to get a positive signal detection of Gag specific CD8+ T cells. The positivity cut-off, shown as the red horizontal line, was set at 0.001% which was double the highest frequency of the HIV-1 seronegative donors. Of the seven VCs we assayed, six had detectable Gag-specific class II-restricted CD8+ T cells on at least one draw date while only VCs AA, AD and AF had detectable class II-restricted CD8+ T cells at more than one draw date. VC AA and VC AD had the most detectable class II-restricted CD8+ T cells on most of the draw dates we tested. Two of the four patients enrolled into the study as chronic patients, had detectable class II-restricted CD8+ T cells. The frequency of the Gag-specific class II-restricted CD8+ T cells observed in the chronic patients was much lower than the frequency observed in the VC patient cohort. Notably, no class II-restricted CD8+ T cell were detected in all our five HLA-matched seronegative donors. Single time points were assayed for the chronics and seronegative donors due to limited longitudinal sample availability. *Two draw dates when the patient VCAA was on ART.

Using tetramer staining of cryopreserved PBMCs, the five available HIV-1 seronegative donors did not have detectable HLA DRB1-restricted Gag_293-312_–specific CD8+ T cells (Figs 3B and 4). Two of the four available HIV-1+ chronic patients had a very low frequency of HLA DRB1-restricted Gag_293-312_–specific CD8+ T cells while the remaining two patients did not have any detectable HLA DRB1-restricted Gag_293-312_–specific CD8+ T cells (Figs 3C and 4). The HLA DRB1-restricted Gag_293-312_–specific CD8+ T cells in the chronic patients were predominantly effector memory cells, 62.5% of the HLA class II-restricted CD8+ T cells for patient 425 and 93.3% for patient 830 (Fig. 3D). Of the seven VC patients tested, only two, VC AA and VC AD, had consistent detectable HLA DRB1-restricted Gag_293-312_-specific CD8+ T cells across multiple visit dates during the course of their infection (Figs 3E and 4, Supp Figs 3A and 4A). These HLA DRB1-restricted Gag_293-312_–specific CD8+ T cells were predominantly central memory (CM) and effector memory (EM) phenotypes (VCAA = CM: 15.3-40.5%, EM: 43.6–67.2%) (VCAD = CM: 34.3–43.9%, EM: 22_44.4%) (Fig. 3F, Supp Figs 3B and 4B). We did not see any correlation between the estimated length of infection and frequency of class II-restricted CD8+ T cells or correlation between CD4+ T cell count and frequency of class II-restricted CD8+ T cells. In summary, two natural HIV-1 virus controllers had HLA DRB1-restricted Gag_293-312_–specific central and effector memory CD8+ T cells consistently present at low frequencies (0.0011–0.018%).

### HLA DRB1*0701 restricted Gag_293-312_–specific CD8+ T cells are a unique T cell subset that expresses both CD4+ and CD8+ T cell-specific genes

We next asked whether these unconventional HIV-1 class II CD8+ T cells have a unique transcriptional profile compared to conventional class I CD8+ T cells and conventional class II CD4+ T cells. The very low frequency of class II- CD8+ T cells (barely at the limit of quantitation) in two of the four chronic patients tested and their absence in the healthy donors (Fig. 4) limited our gene expression analysis to the two VC patients, VCAA and VCAD, that had the highest frequencies and most consistent (across multiple time points) class II- CD8+ T cells. Gag-specific class II-restricted CD4+ T cells, Gag-specific class II-restricted CD8+ T cells and Gag-specific class I-restricted CD8+ T cells were stained, and flow sorted with class I or class II fluorophore-labeled tetramers (Supp Fig. 5) and RNA-sequencing performed on bulk sorted tetramer positive cell populations. The purity of the sorted CD4 and CD8 T cell subsets ranged from 97.1–98.9%.

**Figure 5:**
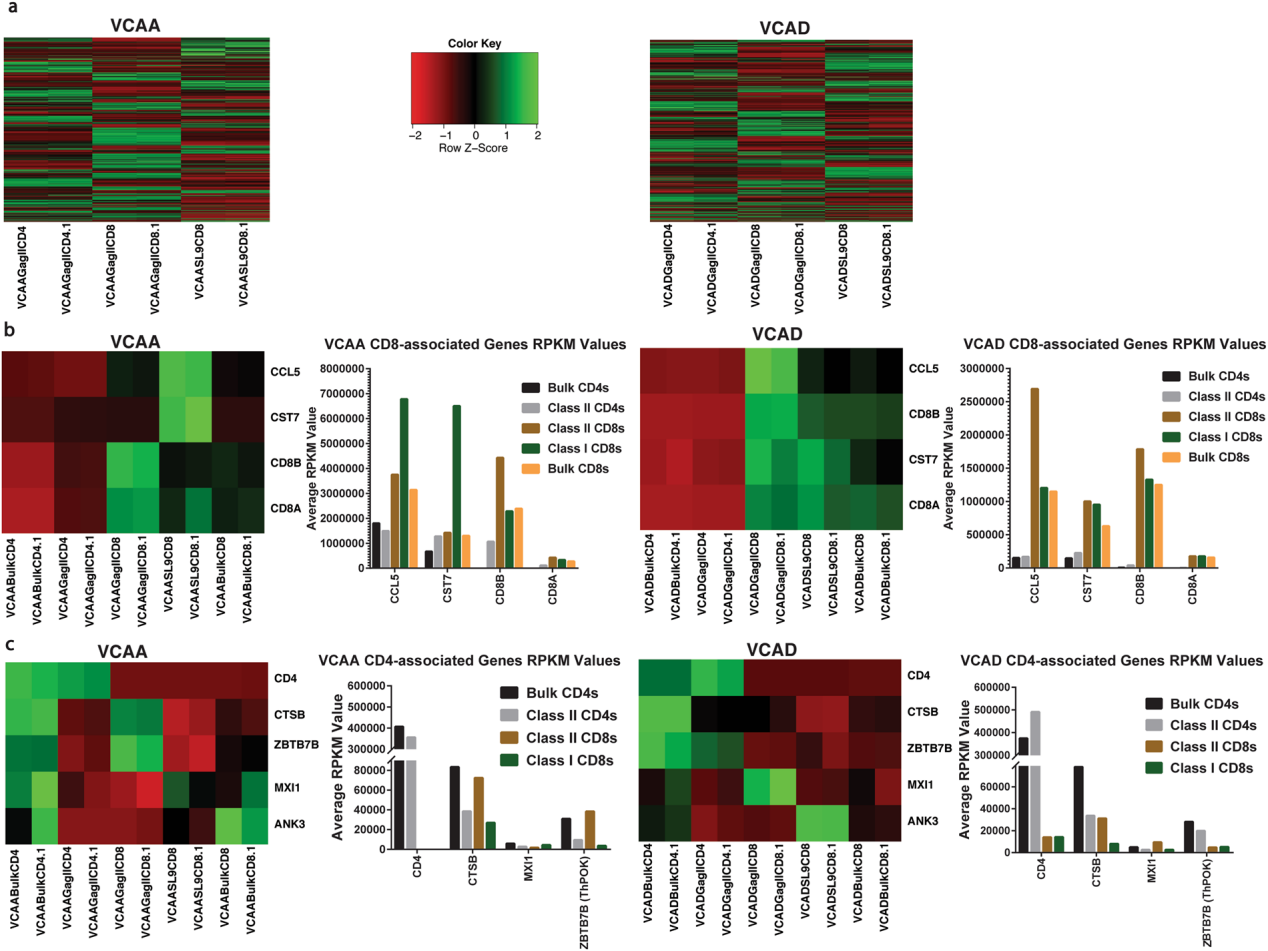
(**A**). Gag-specific HLA DRB1*0701 restricted CD8+ T cells have a unique gene expression profile. Heatmap and hierarchical clustering of significant differentially expressed genes in tetramer sorted Gag-specific class II-restricted CD4+ T cells (GagIICD4), class II-restricted CD8+ T cells (GagIICD8), and class I-restricted CD8+ T cells (SL9CD8) from VCAA (5100 genes) and VCAD (4886 genes). Two replicates are shown for each subset. Green represents high expression, while red represents low expression. (**B**) Gag-specific HLA DRB1*0701 restricted CD8+ T cells express CD8+ T cell lineage-specific genes. Heatmap comparing gene expression levels of CD8+ T cell lineage specific genes CCL5, CST7, CD8A and CD8B in bulk sorted CD4+ T cells (BulkCD4), tetramer sorted Gag-specific class II-restricted CD4+ T cells (GagIICD4), class II-restricted CD8+ T cells (GagIICD8), class I-restricted CD8+ T cells (SL9CD8) and bulk sorted CD8+ T cells (BulkCD8) from VCAA and VCAD. Two replicates are shown for each subset. Green represents high expression, while red represents low expression. The bar plot shows the average reads per kilobase million (RPKM) values of the target genes. (**C**) Gag-specific HLA DRB1*0701 restricted CD8+ T cells express CD4+ T cell lineage-specific genes. Heatmap comparing gene expression levels of CD4+ T cell lineage specific genes CD4, CTSB (Cathepsin B), ZBTB7B (ThPOK), MXI1 and ANK3 in bulk sorted CD4+ T cells (BulkCD4), tetramer sorted Gag-specific class II-restricted CD4+ T cells (GagIICD4), class II-restricted CD8+ T cells (GagIICD8), class I-restricted CD8+ T cells (SL9CD8) and bulk sorted CD8+ T cells (BulkCD8) from VCAA and VCAD. 2 replicates are shown for each subset. Green represents high expression, while red represents low expression. The bar plot shows the average reads per kilobase million (RPKM) values of the target genes.

HLA class II-restricted CD8+ T cells (GagIICD8) in both patients had a unique gene expression profile compared to HLA class I-restricted CD8+ T cells (SL9CD8) and HLA class II-restricted CD4+ T cells (GagIICD4) (Fig. 5A) (Table S1). Given the unique gene expression profile of HLA class II-restricted CD8+ T cells, we then evaluated the expression of known human CD4+ and CD8+ specific genes and lineage-specific transcription factors (Table 4 ^46–48^). Conventional CD4+ T cells are known to be Runx3^L^°^w^ThPOK^High^, while conventional CD8+ T cells are known to be ThPOK^L^°^w^Runx3^High^ ^49–51^.

**Table 4:** Gene signatures used to differentiate human CD4 and CD8+ T cells^46,47^.

CD8+ T Cell Gene Signature	CD4+ T Cell Gene Signature
CD8α, CD8β, CCL5, TBX21, CST7	*CD4*, *ANK3*, *MXI1*, *CTSB*, ThPOK (*ZBTB7B*)

CD8+ T cell subsets (BulkCD8 -  CD8+ T fraction composed of both Gag tetramer-specific and Gag tetramer-nonspecific CD8+ T cells, SL9CD8 - Gag p17-specific class I-restricted CD8+ T cells and GagIICD8 - Gag p24-specific class II-restricted CD8+ T cells) all expressed CD8-specific genes (*CD8B*, *CD8A*, *CST7 and CCL5*), which were all absent in the CD4+ T cell subsets (Fig. 5B). Bulk CD4+ T cells and tetramer specific CD4+ T cells (GagIICD4) all expressed *CD4*, while VCAD tetramer specific CD4+ T cells also expressed *ZBTB7B* (ThPOK) and *CTSB* (Fig. 5C). Interestingly, we observed that HLA class II-restricted CD8+ T cells (GagIICD8) also expressed multiple CD4-associated genes, with VCAA class II CD8s expressing *ZBTB7B* (ThPOK) and *CTSB*, while VCAD class II CD8s expressed *MXI1* (Fig. 5C) (Table S2). Additionally, Gag tetramer-specific CD4+ T cells (GagIICD4) had reduced expression levels of multiple CD4-associated genes *ThPOK*, *MXI1* and *CTSB* compared to the CD4+ T cell fraction composed of both Gag tetramer-specific and Gag tetramer-nonspecific CD4+ T cells (BulkCD4) (Fig. 5C).

The results from the transcriptomics analysis suggests that HLA class II-restricted CD8+ T cells from patients VCAA and VCAD, while sharing some features from both conventional CD4 and CD8+ T cells, possess some distinct features that make them a unique T cell subset that might possess distinct functional properties that could be harnessed to complement conventional CD8+ T cell-mediated HIV-1 control.

### HLA DRB1*0701 restricted Gag_293-312_–specific CD8+ T cells have an anti-viral gene profile comparable to that of conventional class I-restricted CD8+ T cells

To test the hypothesis that Gag-specific HLA class II-restricted CD8+ T cells have a unique transcriptional profile with evidence of anti-viral gene expression, we profiled expression of known cytolytic molecules and anti-viral genes associated with CD8+ T cell-mediated anti-HIV-1 activity^31,52–55^ (Table 5) in HIV-1 Gag-specific HLA class II-restricted CD8+ T cells, HLA class I-restricted CD8+ T cells and HLA class II-restricted CD4+ T cells from VCAA and VCAD.

**Table 5:** Genes associated with CD8+ T cell-mediated HIV-1 replication inhibition^31,52–55^.

CLASS	GENES
Anti-viral cytokines	*CCL3*, *CCL4*, *CCL5*, *CCL3L1*, *CCL22*, *XCL1*, *CSF2*, *TNFRSF9*, *TNFRSF4*, *IFNγ*, *IL2*
Effector molecules	*Perforin*, *GZMA*, *GZMB*, *GZMH*, *GNLY*, *CD107α*, *GZMK*, *SPON2*, *ITGAL*
Other anti-viral genes	*EOMES*, *TBX21*, *IL21R*, *IL7R*, *IL2RG*, *ID2*, *GATA3*, *IL2RA*

**Figure 6:**
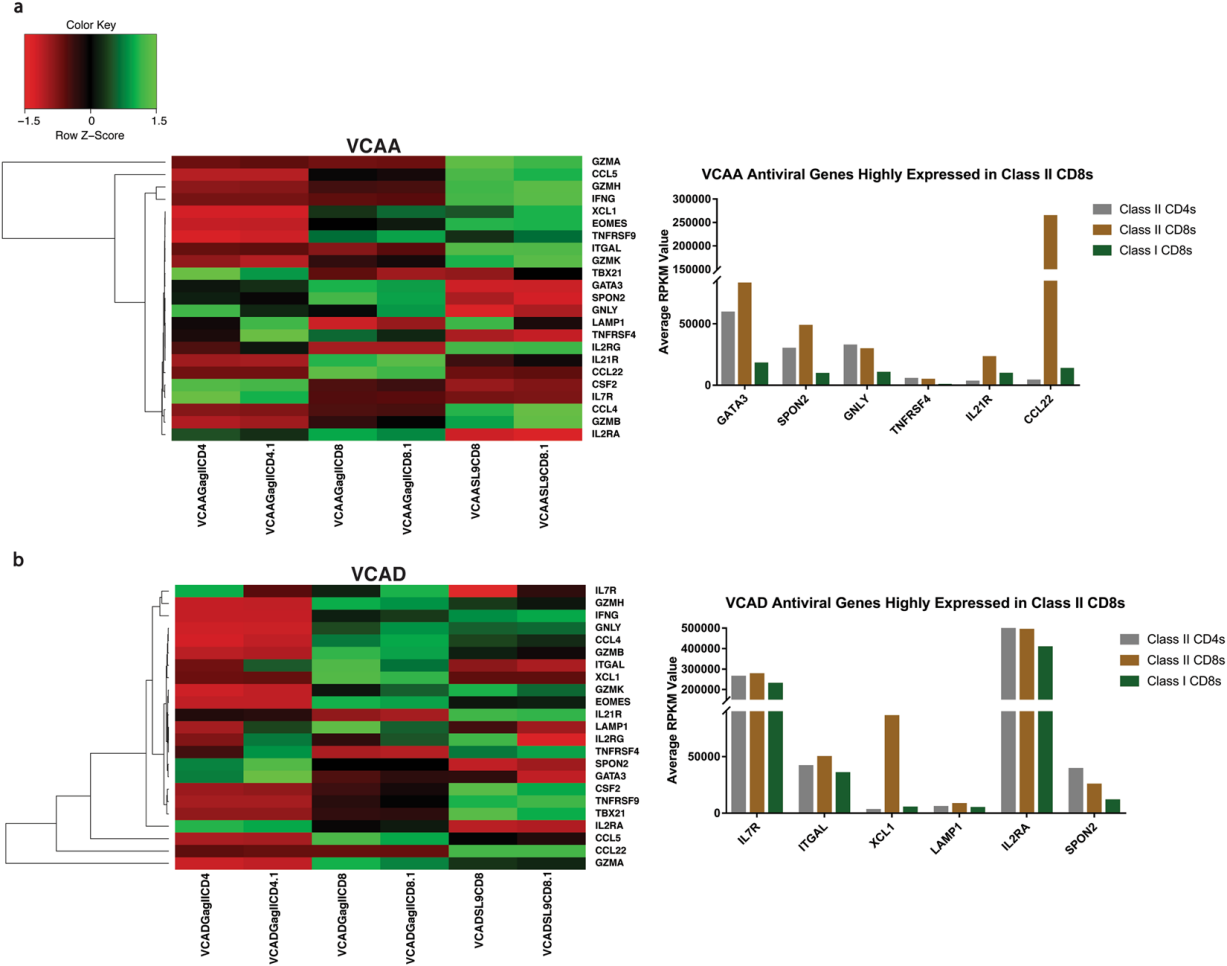
Gag-specific HLA DRB1*0701 restricted CD8+ T cells have a unique anti-viral gene expression profile. Heatmap and hierarchical clustering of anti-viral genes (Table 5) based on expression levels in tetramer sorted Gag-specific class II-restricted CD4+ T cells (GagIICD4), class II-restricted CD8+ T cells (GagIICD8) and class I-restricted CD8+ T cells (SL9CD8) from VCAA (A) and VCAD (B). Two replicates are shown for each subset. Green represents high expression, while red represents low expression. The bar plot shows the average reads per kilobase million (RPKM) values of anti-viral genes of interest highly expressed in class II-restricted CD8+ T cells compared to class I-restricted CD8+ T cells.

**Figure 7:**
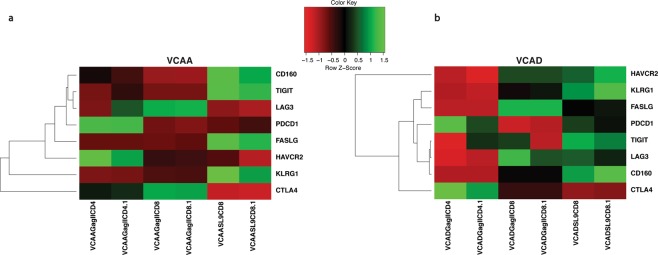
Gag-specific HLA DRB1*0701 restricted CD8+ T cells exhibit a less exhausted expression profile compared to Gag-specific HLA-A*0201 restricted CD8+ T cells. Heatmap and hierarchical clustering of selected T cell exhaustion/activation-related genes (Table 6) based on expression levels in tetramer sorted Gag-specific class II-restricted CD4+ T cells (GagIICD4), class II-restricted CD8+ T cells (GagIICD8) and class I-restricted CD8+ T cells (SL9CD8) from VCAA (**A**) and VCAD (**B**). Two replicates are shown for each subset. Green represents high expression, while red represents low expression.

HLA class II-restricted CD8+ T cells (GagIICD8) in VCAA and VCAD had unique anti-viral gene expression profiles compared to the conventional HLA class I-restricted CD8+ T cells (SL9CD8) (Fig. 6) (Table S2). VCAA HLA class II-restricted CD8+ T cells highly expressed *CCL22*, *IL21R*, *GATA3*, *GNLY*, *IL2RA* and *SPON2* (Fig. 6A), while VCAD HLA class II-restricted CD8+ T cells highly expressed *XCL1*, *ITGAL*, *IL7R*, *LAMP1*, *IL2RA* and *SPON2* (Fig. 6B) compared to their respective HLA class I-restricted CD8+ T cells. *SPON2* and *IL2RA* genes were highly expressed in HLA class II-restricted CD8+ T cells compared to HLA class I-restricted CD8+ T cells in both patients (Fig. 6). We also observed that HLA class II-restricted CD8+ T cells had lower expression of T cell activation and exhaustion markers (Table 6) compared to HLA class I-restricted CD8+ T cells (Fig. 7) (Table S2). VCAA HLA class I-restricted CD8+ T cells highly expressed *CD160*, *TIGIT*, *FASLG* and *KLRG1* transcripts associated with chronic activation and exhaustion (Fig. 7A), while VCAD HLA class I-restricted CD8+ T cells highly expressed *PDCD1* (PD1), *CD160*, *TIGIT* and *KLRG1* transcripts associated with chronic activation and exhaustion (Fig. 7B) compared to their respective HLA class II-restricted CD8+ T cells. *CD160*, *TIGIT* and *KLRG1* were highly expressed in HLA class I-restricted CD8+ T cells in both patients, while *CTLA4* was highly expressed in HLA class II-restricted CD8+ T cells in both patients (Fig. 7). VCAA and VCAD patient-specific variations in anti-viral and exhaustion gene expression profiles were also observed.

**Table 6:** T cell exhaustion-related genes^55^.

Class	Genes
Negative Regulators/Exhaustion Markers	*HAVCR2*, *CTLA4*, *PD1* (*PDCD1*), *TIGIT*, *LAG3*, *CD160*, *FASLG*, *KLRG1*

These results demonstrate that HLA class II-restricted CD8+ T cells in VCs had lower expression of T cell activation and exhaustion markers, and a unique anti-viral gene signature compared to conventional HLA class I-restricted CD8+ T cells.

### DRB1*0701 restricted Gag_293-312_–specific CD8+ and CD4+ T cells have distinct TCR repertoires

To gain an insight into how HLA class II-restricted CD8+ T cells respond to a given antigen, we examined the clonal diversity of HIV-1 Gag-specific HLA class II-restricted CD8+ T cells. Given that we also had HLA class II-restricted CD4+ T cells targeting the same Gag_293-312_ epitope, TCR repertoire analysis enabled measurement of antigen-specific T cell diversity in both HLA class II-restricted CD4 and CD8+ T cells.

**Figure 8:**
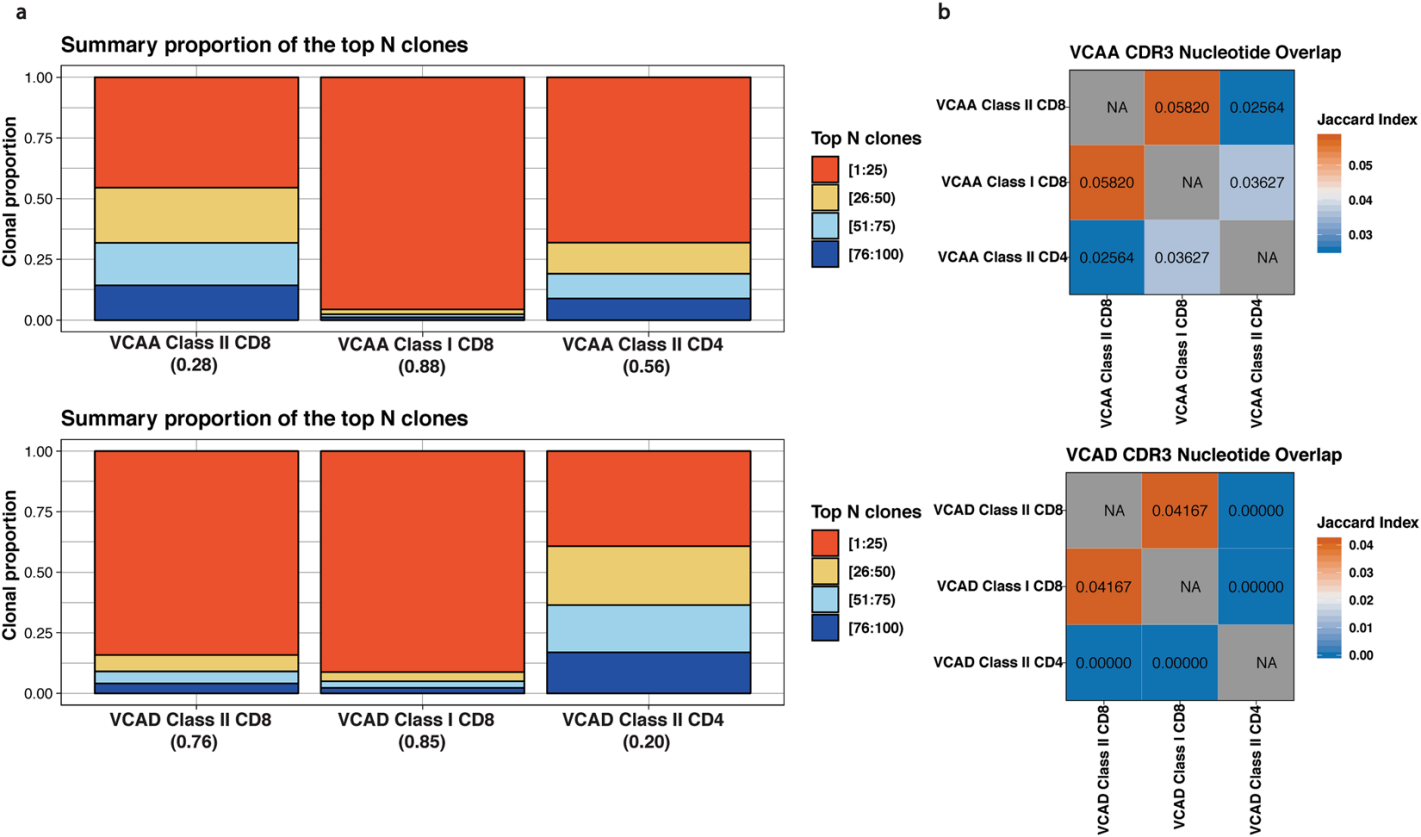
(**A**). TCR repertoire diversity of Gag-specific HLA DRB1*0701 restricted CD4+ T cells, CD8+ T cells and HLA-A*0201 restricted CD8+ T cells in VCAA and VCAD. Top proportions bar plot showing the proportions of the top 100 most abundant clonotypes in repertoires from tetramer sorted Gag-specific class II-restricted CD4+ T cells (GagIICD4), class II-restricted CD8+ T cells (GagIICD8) and class I-restricted CD8+ T cells (SL9CD8) from VCAA and VCAD. The GINI Index calculated by tCR is listed in parentheses below each bar. (**B**) Tetramer sorted Gag-specific HLA DRB1*0701 restricted CD4+ T cells and CD8+ T cells targeting the same epitope have distinct TCR repertoires. Heatmap showing the Jaccard Index evaluating relatedness of TCR repertoires from tetramer sorted Gag-specific class II-restricted CD4+ T cells (GagIICD4), class II-restricted CD8+ T cells (GagIICD8) and class I-restricted CD8+ T cells (SL9CD8) using the TRBV CDR3 nucleotide sequence overlap. The Jaccard Index ranges from 0 (dissimilar) to 1 (similar).

Based on the Gini index which measures the skewness of the clonal distribution (with a low score associated with an equal clonal distribution while a high score is associated with repertoire skewing) from the tcR package^56^, analysis of the top 100 clones in VCAA and VCAD (Table S3) shows that the HLA class I-restricted CD8+ TCR repertoire was consistently narrow and biased in both patients (Fig. 8A), suggesting an oligoclonal expansion of class I-restricted CD8+ T cells in response to Gag p17. For VCAA (no protective alleles), the HLA class II-restricted CD8+ TCR repertoire was broad and diverse while the HLA class II-restricted CD4+ TCR repertoire was narrow and biased (Fig. 8A). VCAD (HLA B57*01 protective allele) had a narrow and biased HLA class II-restricted CD8+ TCR repertoire while the HLA class II-restricted CD4+ TCR repertoire was broad and diverse (Fig. 8A). These findings indicate that there is a polyclonal expansion of HLA class II-restricted CD8+ T cells compared to HLA class II-restricted CD4+ T cells in response to Gag_293-312_ in VCAA, while an oligoclonal expansion of HLA class II-restricted CD8+ T cells compared to HLA class II-restricted CD4+ T cells is observed in VCAD (Fig. 8A).

Given the observed clonal diversity differences between HLA class II-restricted CD8+ and CD4+ T cells targeting the same epitope, we further interrogated the clonal composition of these two subsets by comparing the highly variable Complementarity Determining Region 3 (CDR3) nucleotide sequence overlap. Vβ CDR3 nucleotide sequence overlap across the different T cell subsets, expressed as a similarity Jaccard Index score ranging from zero to one, showed low CDR3 sequence sharing and TCR repertoire similarity between HLA class II-restricted CD8+ and CD4+ T cell subsets (Fig. 8B). In summary, these results demonstrate that HLA class II-restricted CD4+ T cells and HLA class II-restricted CD8+ T cells with shared epitope specificity have distinct TCR repertoires.

## Discussion

CMV-vectored SIV vaccine studies reported control of infection associated with induction of unconventional MHC class E- or II-restricted CD8+ T cells in NHPs^9,11,12^. Studies are ongoing to analyze the specific contribution of MHC-II vs. MHC-Ia vs. MHC-E restricted CD8+ T cell responses to protection in the setting of RhCMV/SIV-mediated prophylactic vaccine protection (Louis Picker, personal communication). Ranasinghe *et al*. first reported that HIV-1-specific cytolytic class II-restricted CD8+ T cells are present in HIV-1 infection^24^. These findings have resulted in increased interest in elucidating the properties of these unconventional class II-restricted CD8+ T cells and their possible role in mediating control of HIV-1 infection. In this study, we assessed the distribution of class II-restricted CD8+ T cells in different HIV-1 patient cohorts, their relative contribution towards CD8+ T cell mediated suppression of HIV-1 replication, ontogeny, functional properties and determined TCR repertoire composition in selected individuals.

To further expand on the work done by Ranasinghe *et al*. and investigate whether atypical class II-restricted CD8+ T cells are only present in HIV-1 VCs, the presence of class II-restricted CD8+ T cells was interrogated in HIV-1 chronic patients, HIV-1 VCs and healthy donors. Among the three HIV-1+ patient cohorts interrogated, we observed that HIV-1+ VC patients had detectable MHC class II-restricted CD8+ T cells, while the frequency of class II-restricted CD8+ T cells were undetectable in the healthy donors and barely at the limit of quantitation in the chronic patients. The majority of VC patients exhibited contact-dependent, non-lytic, anti-viral activity which was abrogated by the addition of anti-MHC monoclonal antibodies, while one patient, VC V, exhibited contact independent non-lytic virus inhibition that was not blocked by the addition of anti-MHC antibodies. We and others have shown that CD8 T cells can mediate contact independent nonlytic virus inhibition^14,30,57,58^, thus potentially explaining why the high CD8+ T cell-mediated viral inhibition activity displayed by VC V was not blocked by the anti-MHC antibodies. Alternatively, VC V CTL activity could be mediated by other non-classical HLA restriction such as HLA-E which has also been reported to mediate CTL activity in humans^59,60^ and non- human primates^10^.

Thus, given our finding and that of others^24^, we believe that natural HIV-1 infection can induce these cells. One hypothesis is that the CD4+ T cell depletion, high viremia and inflammatory cytokine storm present during acute HIV-1 infection creates a CD4 deficient, pro-inflammatory environment^61,62^ that might be conducive for the expansion of these rare class II-restricted CD8+ T cells or their conversion from CD4+ T cells. This possibility is also supported by the observation that CD4-deficient mice mount strong MHC class II-restricted CD8+ T cell responses after primary bacterial or viral infection^15–19^. Additionally, reports from the transplant patients with class II-restricted CD8+ T cells suggested that the highly inflammatory and immunogenic environment might be involved in the induction of these atypical class II-restricted CD8+ T cells^20^. However, more studies are needed to understand the relationship between HIV-1 infection and MHC class II-restricted CD8+ T cells, and further our understanding of additional factors driving or restricting induction and detection of these cells. Moreover, we also observed that the MHC class II-restricted CD8+ T cells present during natural HIV-1 infection had predominant effector and central memory phenotypes. This finding was striking as canonically, CD8+ T cells associated with lower viral loads in chronic HIV-1 infection possess either a central memory^63^ or effector memory phenotype^64,65^ with low PD-1 expression^66,67^.

The presence of a class II-blockable CD8+ T cell mediated anti-viral activity was further supported by the presence of a unique anti-viral gene expression profile in Gag-specific class II-restricted CD8+ T cells. Two anti-viral genes from our down-selected list, *SPON2* and *IL2RA*, were highly expressed in HLA class II-restricted CD8+ T cells compared to HLA class I-restricted CD8+ T cells in both patients. *SPON2* is highly expressed in CD8+ T cells primed to exert effector activities^55,68,69^, while *IL2RA* expression is also required for IL-2 dependent CD8+ T cell effector responses^70,71^. *XCL1*, which was highly expressed in VCAD HLA class II-restricted CD8+ T cells inhibits a broad range of HIV-1 isolates, independent of viral co-receptor usage and genetic subtype^53^, while *CCL22* which is highly expressed in VCAA class II-restricted CD8+ T cells is a known anti-viral β-chemokine secreted by both CD8+ and CD4+ T cells during HIV-1 infection^72^.

Potent CD8+ T cell antiviral activity is one of the factors thought to mediate VC status^14,26,27,29,31,32^. Given these findings, it is plausible that the presence of unconventional HLA class II-restricted CD8+ T cells may contribute to the CD8-mediated anti-viral activity present in VCs. MHC class II and E-restricted CD8+ T cells have been reported in the NHP model to have desirable features for potent CD8+ T cell mediated anti-viral activity. Some of these features include a broader antigen epitope recognition profile, unique targeting of supertopes and the ability to target conserved, subdominant epitopes^9^. Thus, it will be interesting to probe whether the HLA class II-restricted CD8+ T cells in VCs and chronic patients have some of the above unique features, and also whether the higher frequency of HLA class II-restricted CD8+ T cells observed in the VC patients might be involved in mediating their superior protective capacity.

Highlighting the ontogeny of these atypical CD8+ T cells is critical for understanding the factors that trigger induction of these cells and hence will provide valuable insight into how these cells can be targeted by vaccines and harnessed to provide protection from HIV-1 or other viral infections. In the NHP study, the rhesus CMV vector was responsible for the induction of MHC class II-restricted CD8+ T cells and not natural infection. However, the observation of unconventional CD8+ T cells in humans in our study and others^20,21,24^, suggests that they might also be induced without the aid of a CMV vector. Preliminary transcriptomic findings from two VC patients, VCAA and VCAD, suggest that HLA class II-restricted CD8+ T cells have a unique transcriptome and express both CD8-associated (*CD8α/β*, *CST7* and *CCL5*) and CD4-associated genes (ThPOK and *CTSB* for VCAA, and *MXI1* for VCAD). This initial observation suggests the possibility that HLA class II-restricted CD8+ T cells could be ‘ex-CD4+ T cells’ that undergo re-programming to adopt CD8+ T cell like features and hence explaining the presence of CD8-specific genes, an anti-viral gene signature usually associated with CD8+ T cells, CD4-specific genes and HLA class II-restriction.

HIV-1 infection has been reported to disrupt CD4+ T cell lineage-defining transcriptional profile^73^. Furthermore, the presence of peripheral lineage-intermediate T cell subsets with CD4 and CD8 surface markers/effector molecule expression patterns in numerous disease settings is not entirely unheard of^74^. Non-traditional CD4+ T cells with cytolytic functions^75–77^, and lineage intermediate CD4+/CD8+ double positive T cell subsets have been previously reported in HIV-1 infection^78,79^. We have also previously reported the presence of anti-viral and cytolytic CD4+/CD8+ double positive T cells in our HIV-1 Virus Controller patient cohort which included patients VCAA and VCAD used in this study^32^. However, the transcriptomics findings are not conclusive due to the limited sample size and also the need for lineage-tracing experiments in murine models to clearly define the relationship between CD4+ T cells and HLA class II-restricted CD8+ T cells. HLA class II-restricted CD8+ T cells exhibit patient-specific transcriptomic features that might be linked to the differences in viral loads, CD4+ T cell counts and other biological differences between patient VCAA and VCAD. It is also possible that these differences might be due to different induction mechanisms of HLA class II-restricted CD8+ T cells in patients VCAA and VCAD.

TCR repertoire clonal diversity analysis highlights repertoire richness and gives an indication of how different T cell subsets respond to a given antigen^80^. TCR diversity enables T cells to respond to multiple foreign antigens^80^ and in the context of HIV-1 infection this will endow the immune system with the ability to recognize circulating epitope variants. However, in the HIV-1 setting, it is not clear whether having a dominant protective CD8+ T cell clonotype or multiple subdominant clonotypes is better for HIV-1 replication control. TCR repertoire diversity of antigen-specific T cells was shown to be comparable between HIV-1-infected and HIV-1-uninfected subjects^81^, while clonal diversity could not distinguish elite controllers from chronic patients with the HLA-B5701 protective allele^82^. However, there are studies showing that HLA class I-restricted CD8+ T cells^82–84^ have narrow and biased repertoires with dominant clonotypes. Narrow and biased repertoires were also recently reported for HLA class II-restricted CD8+ and CD4+ T cells responding to the same HIV-1 Gag antigen in one VC patient^24^.

In our study, HLA class I-restricted CD8+ T cells from both individuals had narrow and biased TCR repertoires suggesting an oligoclonal expansion in response to the Gag p27 antigen. However, the HLA class II-restricted CD8+ and CD4+ TCR repertoires were different in the two individuals even though both T cell subsets were Gag_293-312_-specific and DRB1*0701-restricted. It is interesting to highlight that the one VC patient, VCAD, with a narrow and biased HLA class II-restricted CD8+ TCR repertoire (similar to that of the class I-restricted TCR repertoire) had the protective HLA-B57*01. VCAA, without protective HLA alleles, had a broad and diverse HLA class II-restricted CD8+ TCR repertoire. It is still unclear how the presence/absence of protective HLA alleles would influence HLA class II-restricted CD8+ TCR repertoire diversity as more patients need to be examined to confirm the relationship we observed in our two patients. Despite having different HLA class II-restricted CD8+ TCR repertoires, both patients still met the prespecified VC cohort criteria for enrollment. It remains to be seen how clonal diversity might affect HIV-1 replication control by both HLA class I- and HLA class II-restricted CD8+ T cells.

Even though we were able to detect the rare HLA class II-restricted CD8+ T cells in some of the HIV-1 infected individuals, this study still had a number of limitations. This study utilized the available HLA DRB1*0701 class II-restricted Gag_293-312_ tetramer and is therefore limited to the detection of a small fraction of the entire HIV-1-specific HLA class II-restricted CD8+ T cell subset within each individual and only for those individuals with this matching HLA haplotype. Further studies with more sensitive multimers such as dodecamers instead of tetramers, autologous optimal class II-restricted HIV-1 epitopes (other than the Gag epitope used in this study) within each individual and with other HLA alleles may detect higher frequencies of HLA class II-restricted CD8+ T cells. Further studies to characterize these cells *ex vivo* or through the generation of T cell lines and clones will enable characterization of this cellular subset by probing the class II-restricted breadth (other than Gag_293-312_) and function (functional reactivity to the target HIV-1 peptides, and ability to mediate viral suppression). Limited patient sample availability and very low to no detection of class II-restricted CD8+ T cells in chronic viremics and healthy patients limited investigating the presence of unconventional class II-restricted CD8+ T cells in a larger patient cohort and the comparative RNA seq analysis of patient samples with different disease outcomes. Future studies investigating the frequency and gene expression profile of class II-restricted CD8+ T cells in large HIV-1 patient cohorts with different disease outcomes (acute patients who later progress to become chronic viremics or acute patients who later become virus controllers, and also patients on ART-therapy (treatment-induced controllers) will help assess the importance of these cells in viremic control and also highlight any distinguishing features of these cells in different disease conditions. Additionally, due to the low frequency directly *ex vivo*, we examined expanded CD8 T cells for HLA class II activity. Thus, the expansion process may have impacted the phenotypes of the HLA class II-restricted Gag_293-312_–specific CD8+ T cells. Further studies with more sensitive multimers such as dodecamers instead of tetramers may result in the detection of a larger subset of these cells without prior expansion of the CD8+ T cell subset. However, given these limitations, it is remarkable that HLA class II-restricted CD8+ T cells were detected in these HIV-1 positive individuals and suggests that there might even be a larger subset of these atypical cells that were missed in this study. For the TCR diversity measurements, we were not able to measure the total body TCR diversity due to the complexity of this approach, but we only focused on the TCR diversity of a defined peripheral blood T cell subpopulation at one given time-point during the course of infection. Future studies will focus on utilizing T cells from lymph nodes and blood at multiple time points during course of infection so as get better snapshot of the HLA class II-restricted CD8+ T cells repertoires.

In summary, we have demonstrated the presence of atypical anti-viral memory HLA class II-restricted Gag_293-312_–specific CD8+ T cells with a unique transcriptional profile that includes expression of both CD4 and CD8+ T cell lineage-specific genes in HIV-1 virus controllers (VCs). The presence of these unconventional HLA class II-restricted CD8+ T cells was associated with natural HIV-1 infection since these cells were not present in HIV-1 seronegative individuals. The finding that functional HLA class II-restricted CD8+ T cells are present in HIV-1+ individuals highlights that elucidation of the ontogeny of this activity in humans can lead to a better understanding of how to induce protective CD8+ T cell immunity by vaccination.

## Materials and Methods

### Patient cohorts

Eleven antiretroviral therapy (ART)-naïve HIV-1-infected virus controllers (Table 1) (maintaining plasma HIV-1 loads of <5,000 RNA copies/ml and CD4+ T cell counts of >400 cells/µl) were enrolled through the Adult Infectious Diseases Clinic at Duke University Medical Center. The specimens utilized in this study were restricted to those patients that met this virus controller definition. Although the precise time of infection for these patients is unknown, the minimum length of infection (ranges from >2 to >14 years) is based on when these patients first reported to the clinic (VC M - 8 years, VC O -3 years, VC V – 3 years, VC X – 2 years, VC AA – 12 years, VC AD – 3 years, VC AF – 14 years, VC AG – 4 years, VC AH – 13 years, VC AJ – 4 years and VC AX – 11 years). Four HIV-1+ chronic patients (Table 2) and five healthy uninfected donors (Table 3) were enrolled through the Center for HIV/AIDS Vaccine Immunology and Immunogen Discovery (CHAVI-ID). All patients were HLA typed using the next generation sequencing typing method. PBMC genomic DNA underwent PCR amplification and cDNA library preparation before allelic-level 3^rd^ generation sequencing typing of full-length HLA genes was performed using the Illumina platform (ProImmune Ltd, Oxford, UK). Nine VCs were evaluated in the Viral Inhibition Assays (VIA) measuring the ability of CD8+ T cells to mediate suppression of multiple HIV-1 virus strains, while seven of the nine VCs from the VIA (based on sample availability) were tested for the contribution of HLA II-restriction in CD8+ T cell-mediated anti-viral activity. Sample availability and HLA haplotype (DRB1*0701) determined which samples (VCs, chronic viremics and healthy donors) were further characterized with HLA tetramers by flow cytometry. Frequency of class II-restricted CD8+ T cells and their presence at multiple time points in one patient determined which patient samples were evaluated by RNA-seq analysis. The studies were reviewed and approved by the Duke University Medical Center Institutional Review Board, and all participants provided written informed consent. All research was performed in accordance with the Duke University School of Medicine guidelines and regulations.

### PBMC isolation and storage

Peripheral blood mononucleated cells (PBMCs) were isolated from whole blood using the Ficoll-Plaque Plus (GE Healthcare) density gradient centrifugation. PBMC were frozen (90% fetal bovine serum [FBS]-10% dimethyl sulfoxide) and stored in liquid nitrogen until analyzed.

### Cell culture and cell subset preparation

Cryopreserved PBMCs were thawed in a 37 °C water bath, transferred to prewarmed RPMI 1640 (Gibco/Invitrogen) supplemented with 10% heat-inactivated FBS and 1% penicillin/streptomycin (Invitrogen) and then washed 2 times before they were examined for recovery and viability. 1 million cells per mL were activated by 50 ng/mL anti-CD3 (eBioscience) and 100 ng/mL anti-CD28 antibodies for 7 days in media containing RPMI 1640 supplemented with 20% heat-inactivated FBS, 1% penicillin-streptomycin, and 20 U/ml recombinant human interleukin-2 (IL-2) (PeproTech). For generating effectors and targets for the virus inhibition assay (VIA), activated PBMCs were separated into CD8+ and CD8− subsets after 3 days in culture using a CD8+ T cell isolation kit and CD8+ depletion beads, respectively (Miltenyi Biotech).

### MHC class II and MHC Class I tetramers

HLA DRB1*0701 Gag_293-312_ tetramers (FRDYVDRFYKTLRAEQASQE), HLA DRB1*0701 Human CLIP_87-101_ tetramers (PVSKMRMATPLLMQA), and HLA-A*02:01 SL9 Gag_77-85_ tetramers conjugated to either PE or BV421 were obtained from the NIH Tetramer Facility (Atlanta, GA). The tetramers were used at a final concentration of 10 µg/ml.

### Cell staining and flow cytometry

On day 7 of cell culture, activated PBMCs were enriched for CD8+ T cells using a CD8+ T cell isolation kit (Miltenyi Biotech) and then examined for viability. 8 × 10^6^ CD8+ T cells were stained with a combination of either 10 µg/ml HLA DRB1*0701 Gag_293-312_-PE and 10 µg/ml HLA DRB1*0701 Gag_293-312_-BV421 tetramer or 10 µg/ml HLA DRB1*0701 Human CLIP_87-101_-PE and 10 µg/ml HLA DRB1*0701 Human CLIP_87-101_-BV421 tetramers (NIH Tetramer Facility) for 1 hr at room temperature (or 30 minutes at 37 °C for the HLA-A*02:01 SL9 Gag_77-85_ tetramers). The cells were then washed once with PBS/2% FBS and then resuspended. The expression of surface molecules was assessed using the following antibodies: CD3-APC-H7 (BD Biosciences), CCR7-BV605 (Biolegend), CD8-FITC (Biolegend), CD56-APC (eBiosciences), CD16-BV711 (BD Biosciences), CD4-AF700 (Biolegend), CD19-PercP-Cy5.5 (BD Biosciences), CD45RA-ECD (Beckman Coulter). The tetramer stained CD8+ T cells were then incubated with the surface marker antibodies for 30 minutes at 4°C in PBS/2% FBS. The cells were washed once with PBS/2% FBS and then stained with Live/Dead Fixable Aqua-Dead Cell Stain (ThermoFisher Scientific, Waltham, MA) for 30 minutes at 4°C in PBS (Invitrogen). The cells were washed once with PBS, and then once with PBS/2% FBS before being fixed in PBS/2% paraformaldehyde. Samples were acquired and analyzed using the LSRII flow cytometer (BD Biosciences) and analyzed using FlowJo software (Tree Star, Ashland, OR). For RNA-sequencing, BD FACS ARIA II (BD Biosciences) was used to sort tetramer stained PBMCs into 350 µL of RLT lysis buffer (Qiagen).

### RNA Sequencing

PBMC-extracted RNA from class II-restricted CD8+ T cells, class I-restricted CD8+ T cells, class II-restricted CD4+ T cells, bulk CD8 and CD4+ T cells from VC AA and VC AD were subjected to reverse transcription and amplification using the Smartseq ultra-low v4 kit (Takara Bio, Mountain View, CA). 200 pg of amplified cDNA was prepared for Illumina sequencing using the Nextera XT library preparation kit (Illumina, San Diego, CA). Libraries were quantified using qPCR (Kapa Biosystems, Wilmington, MA) and sequenced to a minimum depth of 25 million reads per sample (2 × 75 bp reads) on the Illumina NextSeq (NCBI Sequence Read Archive Accession number: PRJNA542922). Raw FASTQ files were merged and then trimmed using the default settings of the cutadapt Python module (http://cutadapt.readthedocs.io/en/stable/index.html). The trimmed reads were aligned to the human genome GRCh38 using STAR^85^. After reference alignment read counts on each gene were quantified by HTSeq^86^, significantly differentially expressed transcripts were determined by DESeq2 R package^87^, and filtering for adjusted p values of less than 0.05 as seen in Fig. 5A. For Fig. 5B through 7, heat maps were generated by comparing the Reads Per Kilobase of transcript per Million mapped reads (RPKM) using lists of known associated genes and calculating the z-scores by row.

Gene Network analysis was done using GeneMania^88^ and pathway analysis was performed using Ingenuity Pathway Analysis (QIAGEN Inc., https://www.qiagenbioinformatics.com/products/ingenuity-pathway-analysis). MiXCR, an open source immune repertoire analysis package (https://github.com/milaboratory.mixcr), was used for paired-end read merging and extraction of TCR sequences from bulk RNA-seq data^89^. TCR repertoire analysis was then performed using the open source software tool tcR (https://github.com/imminfo/tcr)^56^.

### Transmitted/founder virus

Replication-competent virus stocks from full-length infectious molecular clones (IMCs) expressing transmitted/founder viruses (CH058.c, WITO, WEAU3, CH040 and CH077) and lab-adapted NL4.3 were generated as described^14,90^. Briefly, proviral DNA was transfected into 293T cells by using Fugene HD (Roche). Working stocks were amplified by passaging virus in human PBMC (American Red Cross). PBMC-derived virus supernatants were collected every 2 to 3 days and filtered through a 0.45 µm syringe filter, and titers were determined on TZM-bl cells (obtained through the NIH AIDS Research and Reference Reagent Program, Division of AIDS, NIAID, NIH, from J C. Kappes, Xiaoyun Wu, and Tranzyme, Inc.)^14.^

### Viral inhibition assay

This assay to measure the ability of CD8+ T cells to suppress HIV-1 replication in infected target CD4+ T cells has been previously described^14^. Briefly, CD4+ enriched target cells were infected with HIV-1 infectious molecular clones (MOI = 0.1) via 2-hour spinoculation at 1200 RPM. Following spinoculation, infected target cells were plated at 2.25 × 10^4^ cells/well and CD8+ effector T cells were added to cultures at varying Effector:Target ratios (0.25:1, 0.5:1, 1:1, 2:1). Co-cultures were incubated at 37 °C and 5% CO_2_ for three days and virus production was measured by TZM-bl assay^14^ with a readout of relative light units (RLU).

### Blocking assays

This assay was adapted from the previously described virus inhibition assay^14^ to measure the ability of CD8+ T cells to suppress HIV-1 infection by MHC Class I and/or Class II recognition of infected target cells. Briefly, CD4+ enriched target cells were infected with HIV-1 infectious molecular clones (MOI = 0.1) via 2-hour spinoculation at 1200 RPM. Following spinoculation, infected target cells were plated at 2.25 × 10^4^ cells/well and incubated for 30 minutes in the presence or absence of monoclonal antibodies targeting MHC Class I (3F10), MHC Class II (L243), or a negative isotype control (P3x63). Following the incubation, CD8+ effector T cells were added to cultures at varying Effector:Target ratios (0.125:1, 0.25:1, 0.5:1, 1:1). Co-cultures were incubated at 37 °C and 5% CO2 for three days and virus production was measured by TZMbl assay^14^ with a readout of relative light units (RLU).

### Statistical analyses

Statistical significance of results was determined by the non-parametric Sign test performed using SAS (r) Proprietary Software 9.4 (TS1M2), Copyright (c) 2002–2012 by SAS Institute Inc., Cary, NC, USA, and the graphs were generated using GraphPad Prism 8.

## Supplementary information

Supplementary Information - HLA class II-Restricted CD8+ T cells in HIV-1 Virus Controllers

Supplementary Table S1

Supplementary Table S2

Supplementary Table S3

## Data Availability

The RNA-seq datasets generated during and/or analyzed during the current study are available in the NCBI Sequence Read Archive repository (SRA), Accession number PRJNA542922. All data needed to evaluate the conclusions in the paper are present in the paper and/or the Supplementary Materials. Additional data are available from the corresponding author on reasonable request.
