# Supplementary material for: HLA class II-Restricted CD8+ T cells in HIV-1 Virus Controllers
Source: Sci Rep. 2019 Jul 15;9:10165. doi: 10.1038/s41598-019-46462-8 (PMC6629643; doi:10.1038/s41598-019-46462-8)
Supplement: Supplementary file 1 — Supplementary Information - HLA class II-Restricted CD8+ T cells in HIV-1 Virus Controllers [file 41598_2019_46462_MOESM1_ESM.pdf]

Supplementary Information

HLA class II-Restricted CD8+ T cells in HIV-1 Virus Controllers

Tinashe E. Nyanhete[1,2+], Alyse L. Frisbee[1+], Todd Bradley[1,3], William J. Faison[1,3], Elizabeth Robins[2], Tamika Payne[1,4], Stephanie A Freel[1], Sheetal Sawant[1], Kent J. Weinhold[2,4], Kevin Wiehe[1,3], Barton F. Haynes[1,2,3], Guido Ferrari[1,3,4], Qi-Jing Li[2], M. Anthony Moody[1,2,6], Georgia D. Tomaras*[1,2,3,4,5]

Supplementary Fig 1.

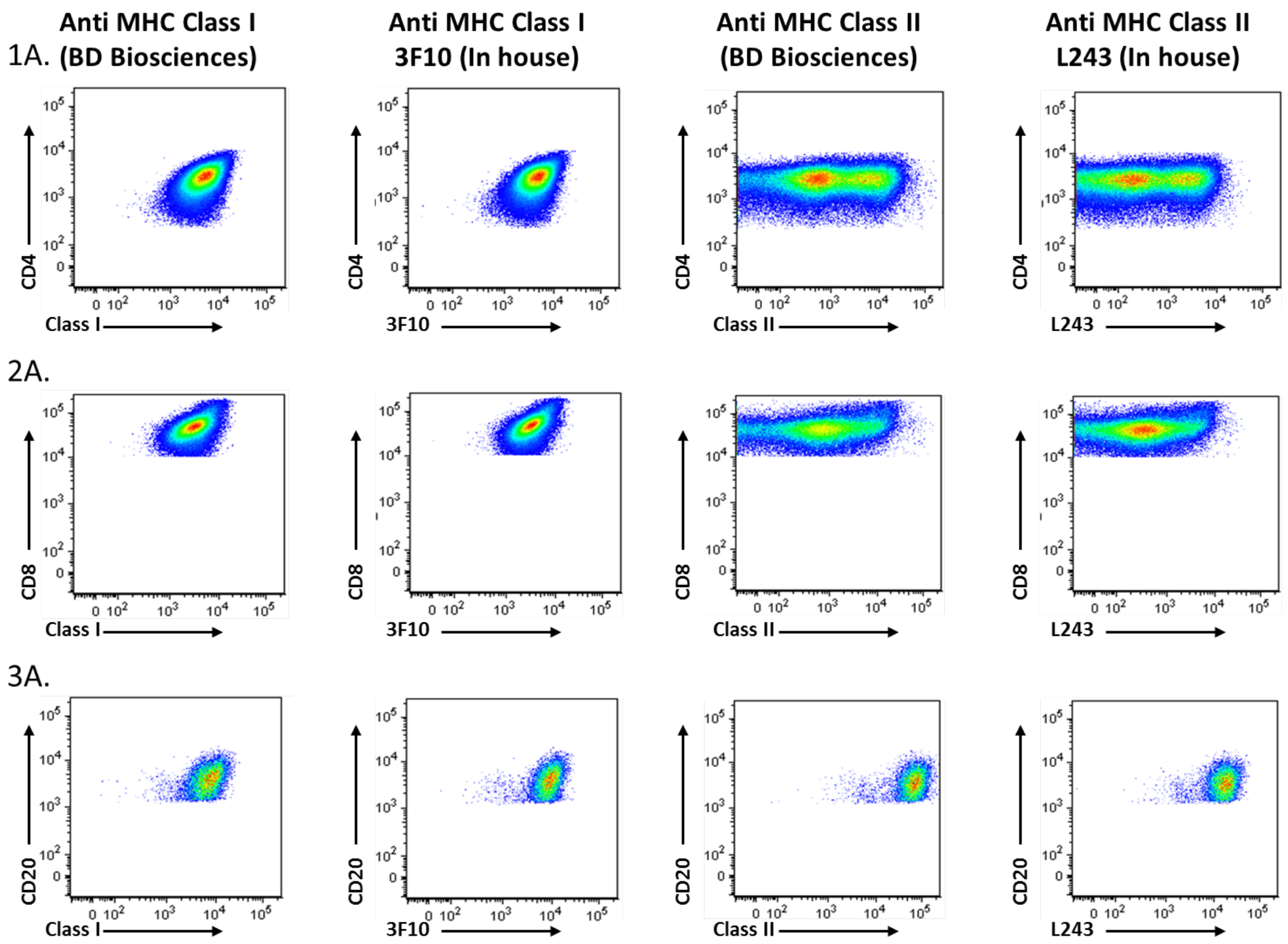

Supplementary Figure 1. In house anti-MHC Class I and anti-MHC Class II antibodies specificity was confirmed as compared to their commercial derivatives in CD4+ T cells (1A); CD8+ T cells (1B) and CD20+ B cells (1C).

Supplementary Fig 2A.

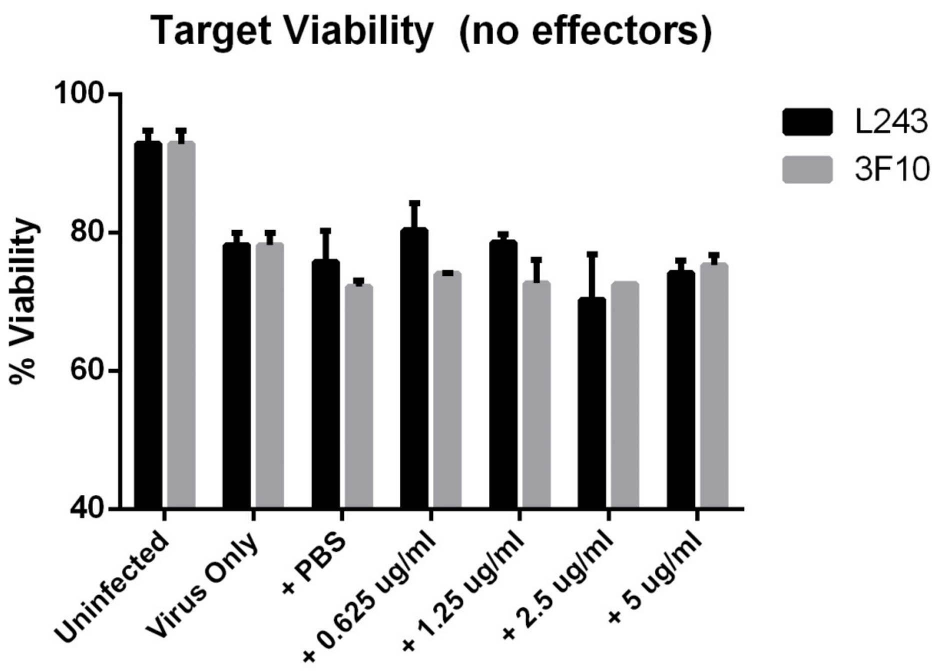

Supplementary Fig 2B.

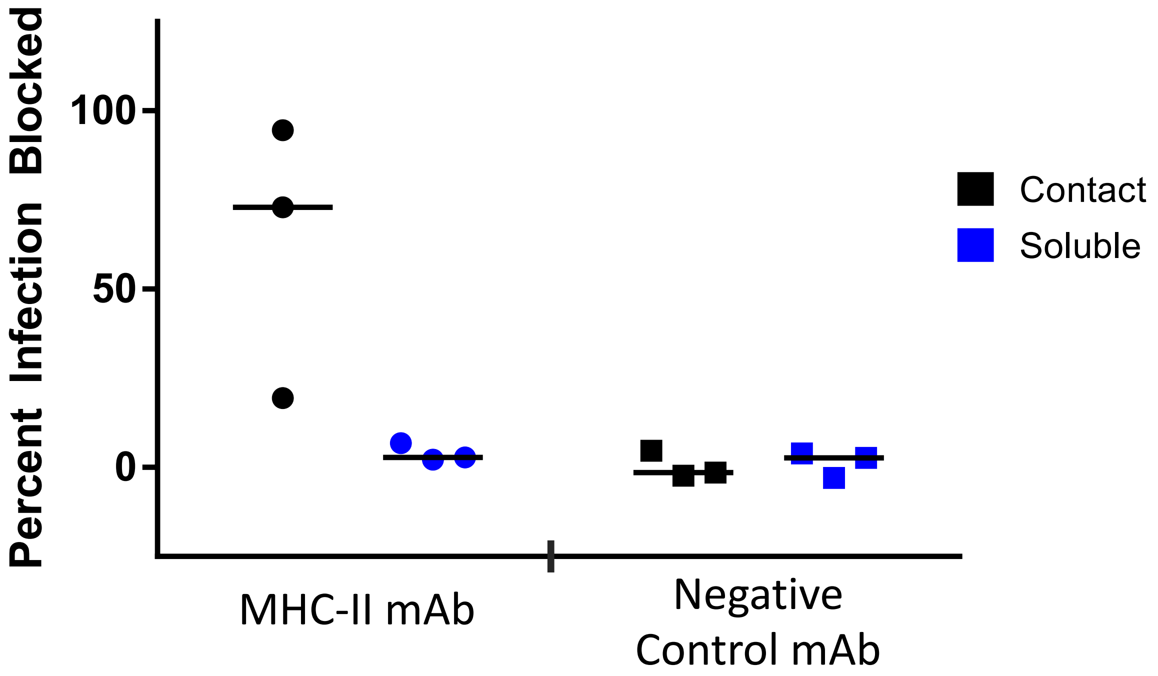

Supplementary Figure 2A. VCAH CD4+ T cell viability (in the absence of effectors) with serial dilutions of anti-MHC Class I (3F10) or anti-MHC Class II (L243) blocking antibody treatments. The blocking antibodies did not have notable effects on viability relative to the vehicle control (PBS only).

Supplementary Figure 2B. MHC blocking of CD8 antiviral activity in CD8+ transwell results in no blocking of viral suppression by CD8+ T cells. VCV CD8 effectors and HIV-1+ infected CD4+ T cells were co-cultured in the presence or absence of MHC antibodies but separated by a semipermeable 0.4μM membrane to eliminate TCR mediated killing. Blocking of CD8 suppression with antibody was not seen when cells were co-cultured in the absence of TCR-MHC contact.

Supplementary Fig 3A.

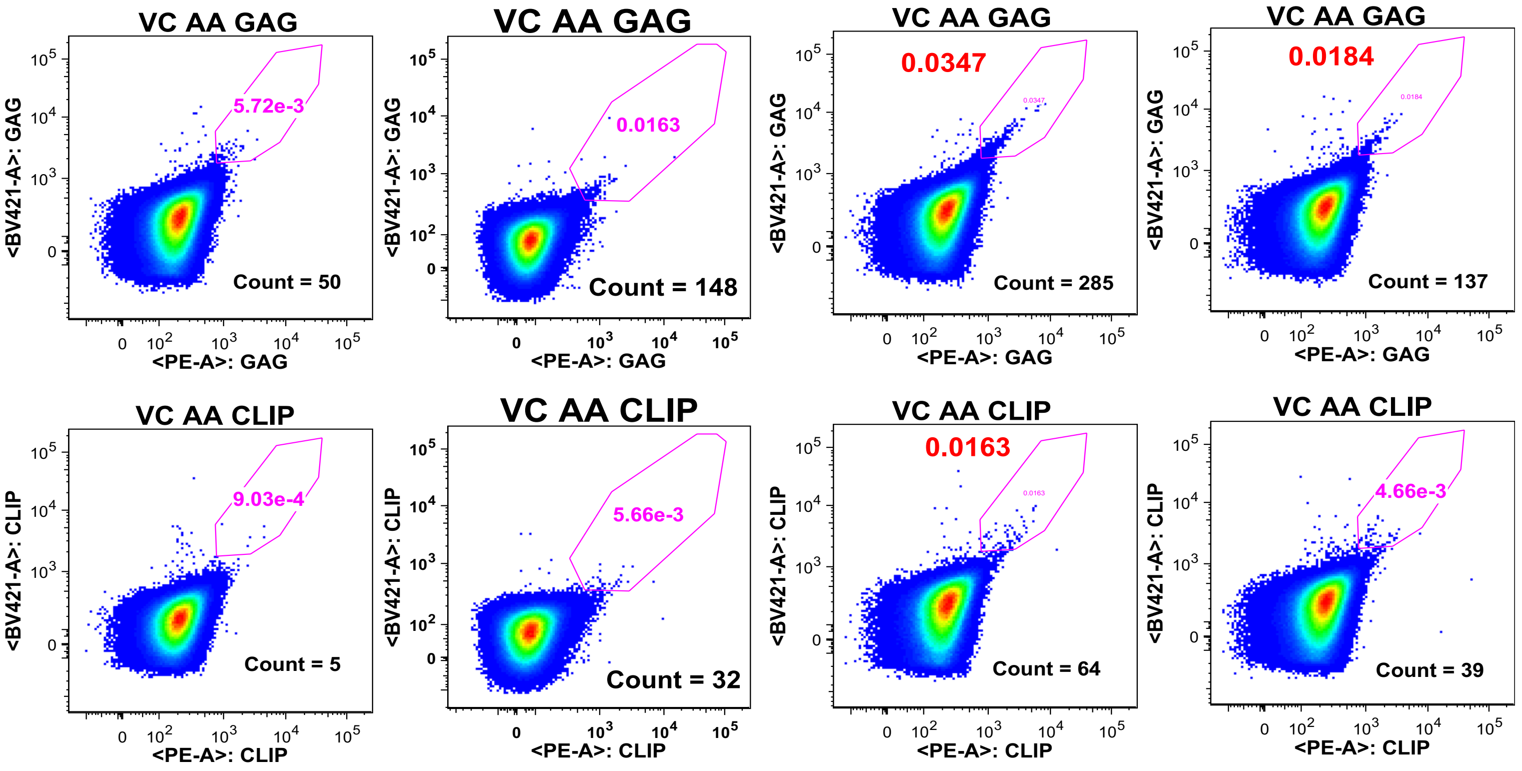

Supplementary Fig 3B.

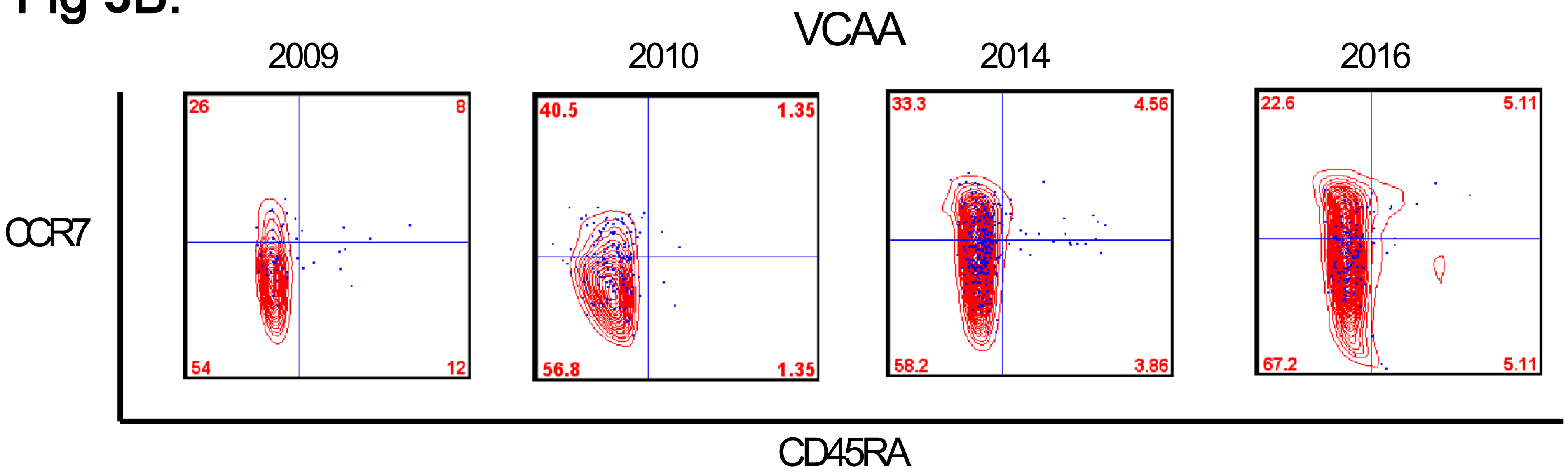

Supplementary Figure 3A. Gag-specific HLA DRB1*0701-restricted CD8+ T cells are present in VC AA at different draw dates. 3B. Gag-specific HLA DRB1*0701-restricted CD8+ T cells in VC AA have predominant effector and central memory phenotypes

Supplementary Fig 4A.

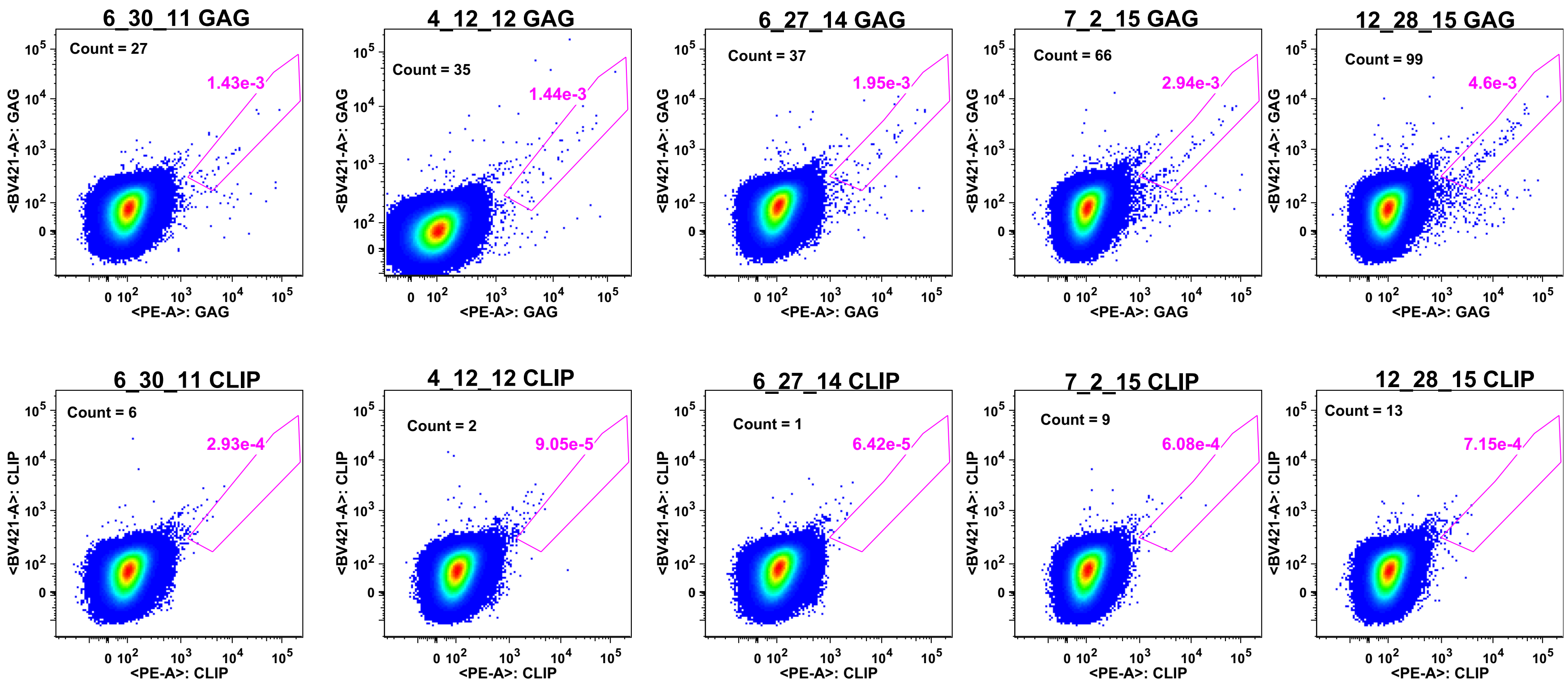

Supplementary Fig 4B.

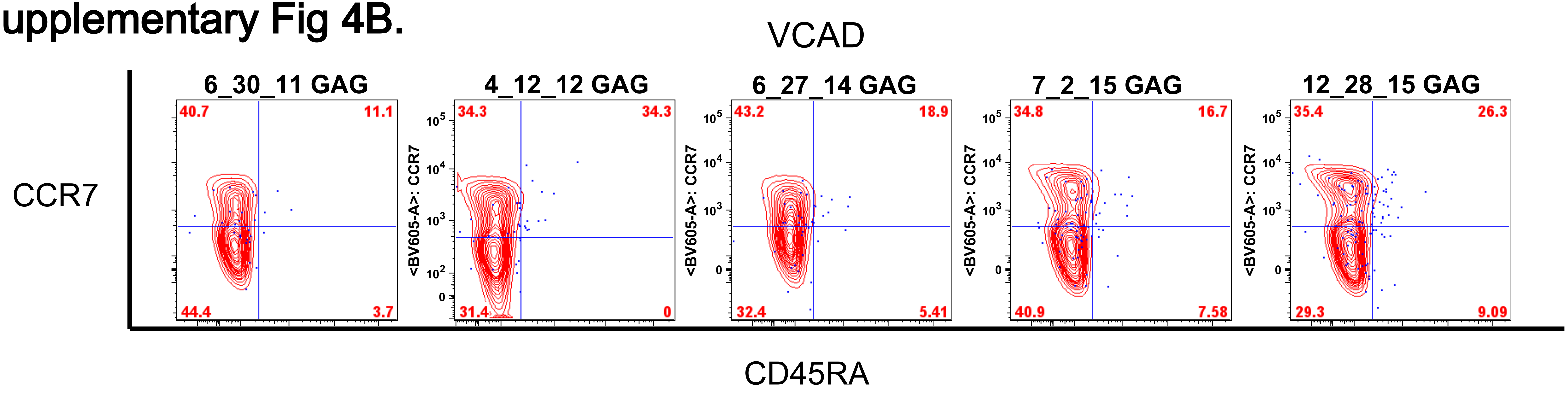

Supplementary Figure 4A. **Gag-specific HLA DRB1*0701-restricted CD8+ T cells are present in VC AD at different draw dates. 4B. Gag-specific HLA DRB1*0701-restricted CD8+ T cells in VC AD have predominant effector and central memory phenotypes**

Supplementary Fig 5.

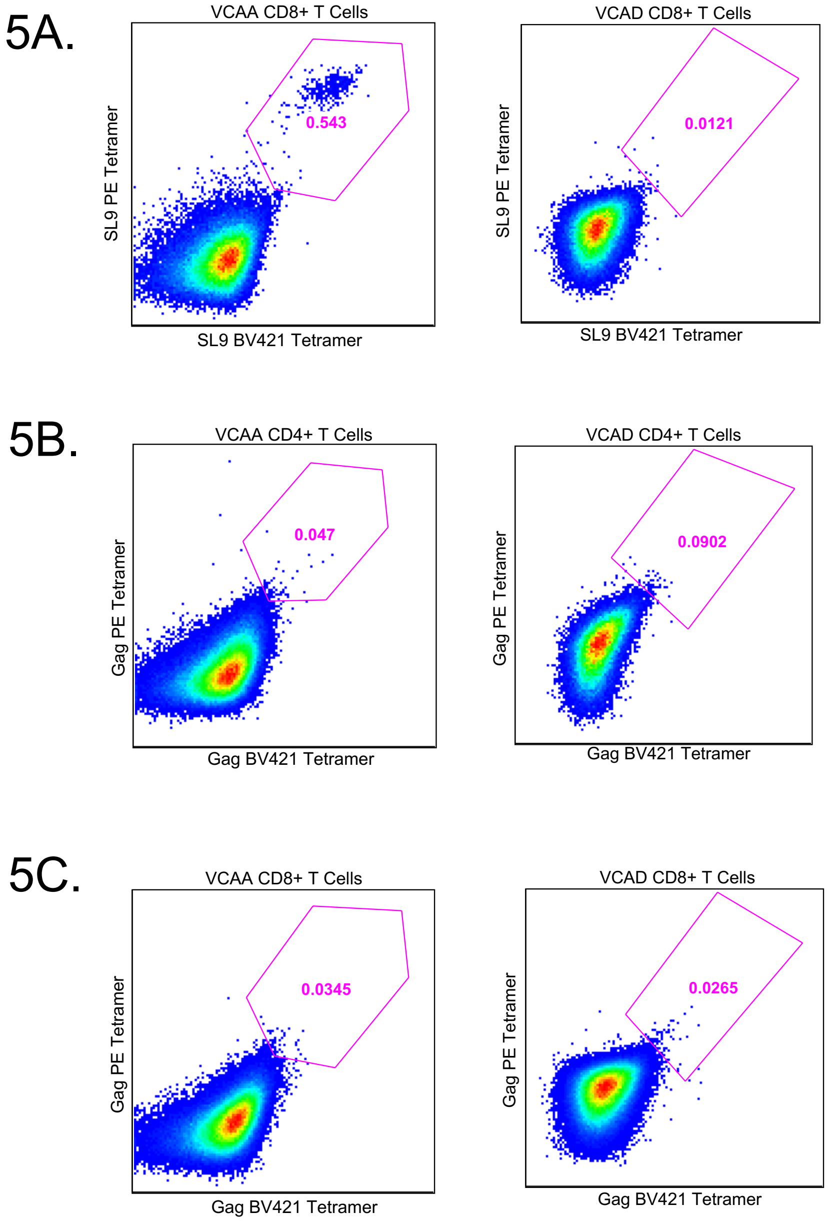

Supplementary Figure 5A. Flow Cytometry sorting gates for the detection and sorting of class I-restricted CD8+ T cells for VCAA and VCAD. 5B. Flow Cytometry sorting gates for the detection and sorting of class II-restricted CD4+ T cells for VCAA and VCAD. 5C. Flow Cytometry sorting gates for the detection and sorting of class II-restricted CD8+ T cells for VCAA and VCAD.
